# Structural Requirements for PACSIN/Syndapin Operation during Zebrafish Embryonic Notochord Development

**DOI:** 10.1371/journal.pone.0008150

**Published:** 2009-12-03

**Authors:** Melissa A. Edeling, Subramaniam Sanker, Takaki Shima, P. K. Umasankar, Stefan Höning, Hye Y. Kim, Lance A. Davidson, Simon C. Watkins, Michael Tsang, David J. Owen, Linton M. Traub

**Affiliations:** 1 Cambridge Institute for Medical Research, University of Cambridge, Cambridge, United Kingdom; 2 Department of Cell Biology and Physiology, University of Pittsburgh School of Medicine, Pittsburgh, Pennsylvania, United States of America; 3 Department of Microbiology and Molecular Genetics, University of Pittsburgh School of Medicine, Pittsburgh, Pennsylvania, United States of America; 4 Institute of Biochemistry I and Center for Molecular Medicine, University of Cologne, Cologne, Germany; 5 Department of Bioengineering, University of Pittsburgh, Pittsburgh, Pennsylvania, United States of America; Thomas Jefferson University, United States of America

## Abstract

PACSIN/Syndapin proteins are membrane-active scaffolds that participate in endocytosis. The structure of the *Drosophila* Syndapin N-terminal EFC domain reveals a crescent shaped antiparallel dimer with a high affinity for phosphoinositides and a unique membrane-inserting prong upon the concave surface. Combined structural, biochemical and reverse genetic approaches in zebrafish define an important role for Syndapin orthologue, Pacsin3, in the early formation of the notochord during embryonic development. In *pacsin3*-morphant embryos, midline convergence of notochord precursors is defective as axial mesodermal cells fail to polarize, migrate and differentiate properly. The *pacsin3* morphant phenotype of a stunted body axis and contorted trunk is rescued by ectopic expression of *Drosophila* Syndapin, and depends critically on both the prong that protrudes from the surface of the bowed Syndapin EFC domain and the ability of the antiparallel dimer to bind tightly to phosphoinositides. Our data confirm linkage between directional migration, endocytosis and cell specification during embryonic morphogenesis and highlight a key role for Pacsin3 in this coupling in the notochord.

## Introduction

Eukaryotic cell excitability and responsiveness typically depend on membrane-embedded surface receptors or channels. Upon ligand binding or activation, these transmembrane proteins can signal to an expansive array of intracellular effectors to regulate, within seconds to minutes, the ion or phosphorylation status of the cell, the activity and positioning of cytoskeletal assemblages and adhesion molecules as well as, over longer time intervals, transcriptional activity. Fine regulation of these types of cellular responses involves precise modulation of the location, intensity and duration of the signaling process. Endocytosis, the removal of select surface macromolecules by internalization within membrane-bounded vesicles, plays an integral part in signaling events. Not only does uptake remove surface receptors from the direct source of soluble ligand, it is now known that different signaling pathways and outcomes can occur from stimulated receptors placed at the plasma membrane or in endosomal carrier vesicles [1]–[4].

Early embryonic development is characterized by extensive cell division followed by remarkable cell migration and reorganization events to generate the basic body plan [5]. The critical cellular movements depend upon complex signaling events, often with extracellular secreted morphogens providing spatially graded signals for individual cells at defined locations within the developing embryo to instruct cell identity and fate determination [6], [7]. Given the essential dependence of early embryonic cell shape changes and coordinated cell movements on signal transduction pathways, and because receptor density, surface half life and localization is impacted by internalization, it seems likely that endocytosis could be importantly involved in normal embryonic development. There is good evidence for this in *Drosophila*, where receptor endocytosis is clearly necessary for productive Notch signaling [2], [8]–[10]. Similar mechanisms operate during development of the zebrafish *Danio rerio*, with ubiquitin-dependent endocytosis of the Notch ligand Delta required for proper Notch signaling [11], [12]. In a different example, long-range tracking of primordial germ cell clusters, which will become the gonad in zebrafish embryos, depends on the chemokine receptor CXCR4 responding to a SDF-1 guidance signal [13]. During the locomotion process, CXCR4 internalization temporarily inhibits directed migration allowing the cells to briefly pause and locally reorient to the chemokine gradient [14]. Also, Dapper2, a zebrafish late endosome-associated protein, directs Nodal-type transforming growth factor-β (TGF-β) receptors toward lysosomal degradation, thereby counteracting mesodermal fate induction [15].

Proper cell migration and positioning in the forming embryo also depends on dynamic remodeling of adhesive cell–cell and cell–matrix contacts. In *Xenopus* embryos, TGF-β family morphogenic ligands induce expression of components that regulate the internalization and recycling of cadherin adhesion molecules [16]. This endocytic activity appears to regulate cell adhesiveness during the morphogenetic changes of early embryogenesis. Analogously, during zebrafish gastrulation, Wnt11 modulates the surface E-cadherin levels in prechordal plate progenitors through a Rab5c-dependent endocytic pathway [17]. Membrane trafficking of E-cadherin appears to facilitate cohesiveness and concerted movement of this defined group of progenitor mesoendodermal cells as an organized group [17]. Accumulating evidence thus implicates endosomes and endosomal regulatory proteins in modulating signaling and adhesion during early embryogenesis.

Here, we focus on clathrin-mediated endocytosis, a process generating the initial vesicular transport intermediates leaving the plasma membrane. PACSIN/Syndapins are EFC (extended FCH (Fes/CIP4 homology)) domain proteins implicated in endocytosis because the C-terminal SH3 domain binds physically to the large GTPase dynamin, the phosphoinositide polyphosphatase synaptojanin, or the branched-actin regulator WASp [18]–[20]. Ectopic expression of PACSINs [21] or the PACSIN 1 SH3 domain [22], [23] interferes with clathrin-dependent internalization. There is also a clear connection between PACSIN/Syndapin and actin cytoskeleton nucleation; overexpression promotes wildly exaggerated filopodia formation [23], [24]. Consequently, it has been posited that PACSIN operates at the intersection between endocytosis and actin assembly [25], [26]. We have solved the structure of the *Drosophila* Syndapin EFC domain (alternatively termed F-BAR due to the gross structural similarity of the EFC and BAR domains [27]) that, together with the recent crystal structures of the human PACSIN 1 and -2 F-BAR domains [28], reveals unique features of this membrane-binding fold. We also identify an unexpected role for the Syndapin-related Pacsin3 in notochord differentiation in *Danio rerio*, and use structure-guided mutagenesis to uncover structural requirements for PACSIN/Syndapin operation *in vivo*. Our data reveal that Pacsin3 influences cellular locomotion to facilitate the columnar organization of the notochord during early development.

## Results

### The Syndapin EFC Domain Structure

**Figure 1:**
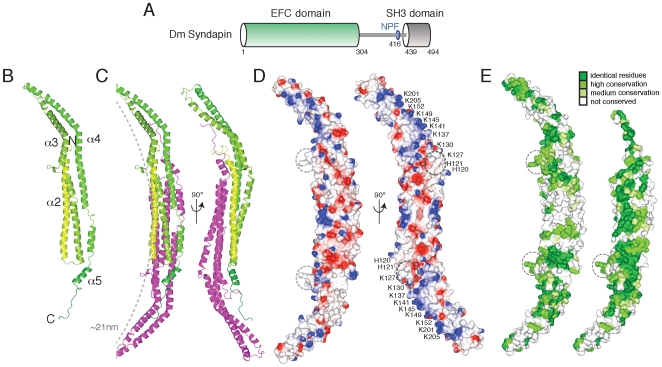
The Syndapin EFC domain structure. (A) Domain organization of *Drosophila* Syndapin. (B) Ribbon diagram of a Syndapin EFC domain monomer colored from light green (N terminus) to dark green (C terminus). (C) The Syndapin EFC dimer in two orientations, with the second monomer colored magenta. The radius of curvature of the EFC dimer (grey dashed arc) is calculated to be ∼21 nm. (D) Electrostatic surface representation of the Syndapin EFC dimer. L123 and M124 (grey dashed circle) protrude from the concave surface, also lined with conserved positively charged Lys/Arg residues and predicted to be the membrane-binding surface. Residues mutated in the 5K→E mutant are shown (bold). (E) Conserved surface representation of Syndapin EFC homologues. Homologues (21, listed in Figure S1) were aligned by MUSCLE [91]. Residues are colored by identity (dark green), high (green), medium (light green) or no (white) conservation. The left panel is similar to *D*, left panel. One monomer is removed (right panel) to reveal the extensive, highly conserved dimer interface. While the concave surface of the Syndapin EFC is conserved across homologues the convex surface is poorly conserved, suggesting the concave surface is functionally important. Surface rendered in CCP4MG [92].

*Drosophila* Syndapin consists of an N-terminal membrane-binding EFC domain (residues 1–304) linked to a C-terminal SH3 domain (residues 439–494) by an unstructured linker including an EH domain-binding Asn-Pro-Phe (NPF) motif (Figure 1A) [26], [29]. Purified full-length Syndapin was used in crystallization trials but the crystals formed contained only a protein corresponding to the predicted N-terminal EFC domain. The structure of this domain (residues 14–301) was solved by X-ray crystallography at 2.7 Å resolution by single-wavelength anomalous dispersion (SAD; Table S1). This EFC domain, like others that have been recently solved (FBP17/CIP4 [30], FCHO2 [31] and PACSIN [28]), is a dimer resembling an elongated bowl when viewed from one side and a tilde-shape from another (Figure 1C). Each monomer (Figure 1B) is composed of three long helices, designated α2 (residues 24–71), α3 (residues 76–118 and residues 127–172) and α4 (residues 182–190, 199–207, 213–253 and 261–273) that are flanked on one end by the N-terminal residues 14–23 and on the other by C-terminal residues 274–301, including a short C-terminal helix α5 (residues 277–288). This nomenclature is consistent with other EFC domain structures that have a short helix (α1) that is either disordered or missing in the Syndapin EFC domain. Monomers dimerize in an antiparallel fashion to form a six-helical bundle core (α2–α4 from each monomer). The Syndapin EFC dimer buries a significant surface area, equivalent to 4295 Å^2^/monomer, similar to the contact area of FBP17 (4765 Å^2^), CIP4 (4020 Å^2^) and FCHO2 (4620 Å^2^). The dimer interface is formed by residues restricted to helices α2 and α4 (Figure 1B). Of the 288 visible residues in the Syndapin EFC domain, 81 participate in the hydrophobic dimer interface and, of these, 66 are strictly conserved in Syndapin homologues. This is clearly seen when Syndapin homologue conservation is mapped onto the EFC structure—the dimer interface shows the most striking degree of conservation (Figure 1E and S1).

### Unique Syndapin Features

**Figure 2:**
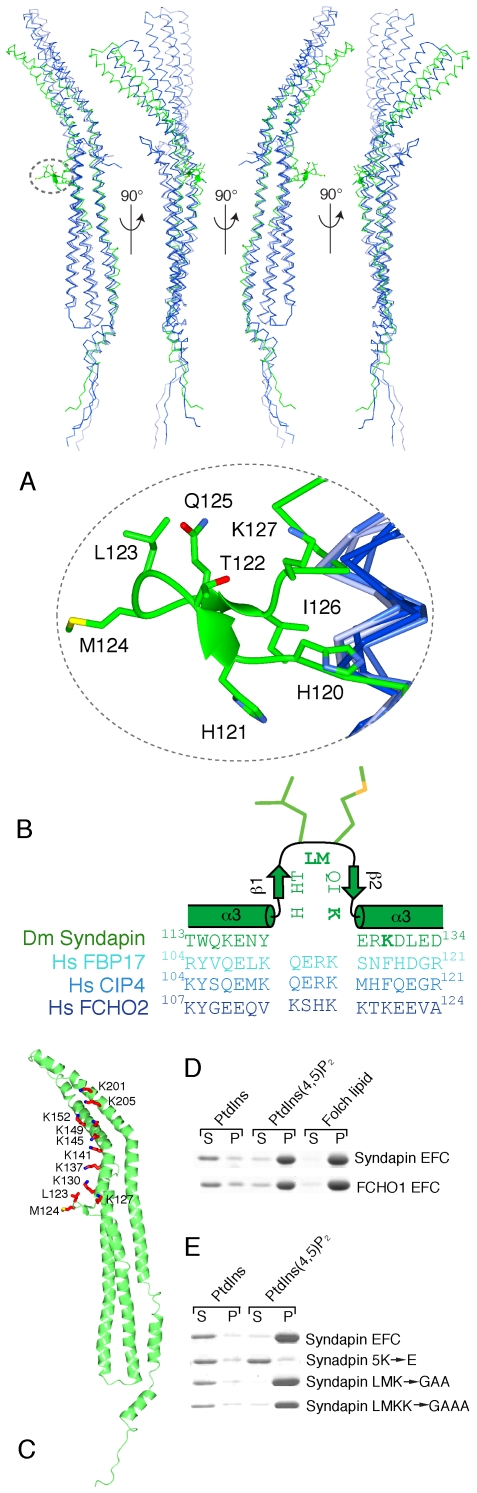
Structural and functional features of the Syndapin EFC domain. (A) Structure-based Cα alignment of Syndapin (green) with the EFC domain structures of FBP17 (light blue), CIP4 (medium blue) and FCHO2 (dark blue). Only the monomers are shown. The unique prong region in the Syndapin α3 is circled in grey above and magnified below. (B) Structure-based sequence alignment of *D. melanogaster* (Dm) Syndapin (green) and the EFC domains of *Homo sapiens* (Hs) FBP17 (light blue), CIP4 (medium blue) and FCHO2 (dark blue) in the vicinity of the unique prong sequence (^120^HHTLMQIK) in Syndapin. L123, M124, K127 and K130 in Syndapin are highlighted in bold. (C) Syndapin EFC domain mutants tested in liposomes binding and/or *in vivo* assays are highlighted in red on the monomer ribbon structure. (D) Syndapin and FCHO1 EFC domains bind synthetic PtdIns(4,5)P_2_ liposomes and Folch extracted brain lipid in sedimentation assays. Regions of Coomassie blue-stained aliquots from supernatant (S) and pellet (P) fractions separated by SDS-PAGE are shown. (E) Syndapin EFC domain mutants binding to liposomes analyzed as in *D*.

Alignment of the EFC domains from Syndapin homologues with other EFC domains including FCHO2 and FBP17/CIP4 reveals two features exclusive to the Syndapin/PACSIN EFC domain. First, α4 in the Syndapin EFC is interrupted by an insert, ^254^DLTKVQS, the ^255^LTK of which assumes a short stretch of 3_10_ helix that packs against the homologous region from the other Syndapin molecule in the dimer. Second, there is an insert of 7–8 residues that is unique to, and conserved in, all PACSIN homologues [28]. The insert in *Drosophila* Syndapin (^120^HHTLMQIK) is structured into a prong that protrudes approximately 18 Å from the concave face of the domain (Figure 2). Residues H121 to T122 and Q125 to I126 form two short β-strands hydrogen bonded into an antiparallel β-sheet scaffold, at the tip of which are positioned two hydrophobic residues, L123 and M124 (Figure 2B). This prong is surrounded by many basic residues that are also conserved in Syndapin homologues (K127, K130, H120, H121, R129, K28, K137, K35, K112; Figure 2C and S1). These features suggest that L123 and M124 insert into the hydrophobic interior of the membrane [28]. Furthermore, as each monomer in the Syndapin dimer has one prong, membrane insertion could occur simultaneously at sites ∼77 Å apart.

### Radius of Curvature of EFC Domains

The closest structural homologue of the Syndapin EFC domain is FCHO2. The two monomers align over 226 Cα atoms with rmsd 3.2 Å. The Syndapin EFC is more distantly related to the EFC domains of FBP17 (181 Cα with rmsd 2.9 Å) and CIP4 (169 Cα with rmsd 3.5 Å). Hence, the overall length of Syndapin and FCHO2 dimers is similar and both shorter than FBP17 and CIP4 because the outer edges of α3 and α4 are splayed laterally (Figure 2A). Yet the radius of curvature of the Syndapin EFC dimer (∼21 nm, assuming prong residues insert into the bilayer) is significantly smaller than CIP4 and FBP17 (both ∼30 nm) or FCHO2 (∼55 nm) (Figure S2) making the packing angle of the Syndapin EFC dimer more similar to the radius of curvature of the N-BAR domains of amphiphysin and endophilin (both ∼11 nm) than to other EFC domains.

### The Syndapin EFC Domain Binds Liposomes

The EFC domains from *Drosophila* Syndapin and FCHO1 associate with synthetic liposomes. Both EFC domains bind to Folch brain extract liposomes as well as to phosphatidylinositol 4,5-biphosphate (PtdIns(4,5)P_2_)- but not phosphatidylinositol (PtdIns)-containing liposomes (Figure 2D). Probing the involvement of the Syndapin prong region, no combination of mutations in residues L123, M124, K127 or K130 (Figure 2E), or in fact deletion of the whole prong has any significant effect on liposome association (Figure S3). The structure of Syndapin EFC is characterized by a continuum of positively charged residues along the concave surface beginning at the center with the Syndapin prong residues K127, K130, H120 and H121 and continuing to the ends with the residues K137, K141, K145, K149, K152, K201 and K205 (Figure 1D and 2C). Introducing negative charges along the concave face of the EFC domain disrupts liposome binding, with the pentamutation K137E, K141E, K145E, K149E, K152E (5K→E) abolishing binding to below detectable levels (Figure 2E) despite maintaining wild-type structure (by circular dichroism). EM analysis of protein-bound liposomes shows the Syndapin EFC domain generates long tubules of two different diameters [28] while the 5K→E mutant has negligible tubulating activity (Figure S3).

To quantify the binding of the Syndapin EFC domain to PtdIns(4,5)P_2_-containing liposomes, and to compare this to other PtdIns(4,5)P_2_-binding endocytic proteins, we used liposome-based surface plasmon resonance [32]. This method is more sensitive than sedimentation assays, allowing concentrations of protein below the *K*_D_ to be used (Figure S3). The endocytic protein epsin 1 binds 200-nm diameter PtdIns(4,5)P_2_-containing liposomes with a *K*_D_ of 590 nM while AP-2 binds with a *K*_D_ of 7.3 µM [32]. The Syndapin EFC domain associates significantly more strongly (*K*_D_ 88 nM) than epsin 1 due to the large electrostatic complementarity between the basic concave surface and the negatively charged convex liposome surface. Disrupting this interaction surface with the 5K→E mutant reduces binding to undetectable levels, whereas simultaneous mutation of L123, M124, K127 and K130 (LMKK→GAAA), reduces binding 14-fold (*K*_D_ 1.2 µM).

### Functional Characterization of PACSIN/Syndapin in Early Development

**Figure 3:**
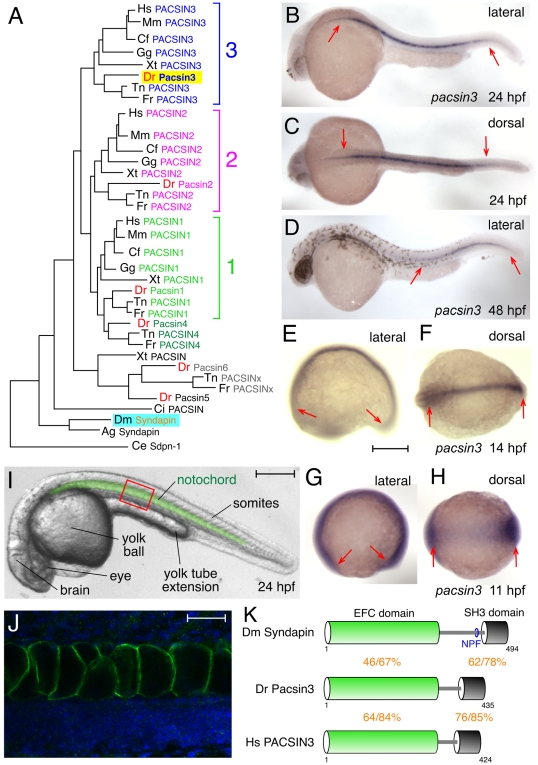
The zebrafish Pacsin3 orthologue. (A) PACSIN family dendrogram (TreeFam accession TF313677). Mm, *Mus musculus*; Cf, *Canis familiaris*; Gg, *Gallus gallus*; Xt, *Xenopus tropicalis*; Dr *Danio rerio*; Tn, *Tetraodon nigroviridis*; Fr, *Fugu rubripes*; Ci, *Ciona intestinalis*; Ag, *Anopheles gambiae*; Ce, *Caenorhabditis elegans*. (B–H) Embryonic *pacsin3* mRNA localization (purple) by whole mount *in situ* with *pacsin3* antisense riboprobe at the various developmental stages noted. Anterior is left. Bar  = 250 µm. (I) Lateral view of a 24 hpf control embryo with the notochord pseudocolored in green to highlight the location of this organ. Other structures apparent at this stage are labeled. Bar  = 250 µm. (J) Indirect immunolabeling (green) of Pacsin3 with affinity-purified antibodies in the notochord at a lateral region of a fixed 24 hpf embryo, analogous to the red box in *I*. Nuclei are counterstained with Hoechst (blue). Bar  = 50 µm. (K) Schematic of the organizational relatedness and domain structural identity/similarity between selected Syndapin/PACSIN isoforms.

*Drosophila* has only a single Syndapin gene and although deletion is semi-lethal at the pupal stage, strong loss-of-function alleles display no overt larval phenotype [29]. Another model system is therefore required for facile structure–function analysis under physiological conditions in a multicellular organism. Chordate genomes typically contain several paralogues; mammals express three PACSIN isoforms, all with the standard EFC–SH3 domain architecture [21], [26], [33]. The zebrafish *Danio rerio* encodes six apparent *pacsin* paralogues (Figure 3) [26], due to teleost fish-specific genome duplication [34]. Whole-mount *in situ* hybridization reveals that during embryonic development, transcripts for the zebrafish *pacsin3* orthologue (zgc:56324) are strongly and highly selectively expressed in the notochord 24 hours post fertilization (hpf); the transcript level decreases and localizes more toward the posterior trunk and tail region at 48 hpf (Figure 3B–D). Localized expression of the zygotic transcript is already evident during segmentation period; at the 3-somite (∼11 hpf) and 14–19-somite stages (16–18 hpf) (Figure 3E–H) [35] and RT-PCR confirms expression of *pacsin3* at the 1-somite stage (not shown). Affinity-purified anti-Pacsin3 antibodies reveal the protein is highly restricted and concentrated on the limiting membrane of expanding notochord cells in 24 hpf embryos (Figure 3I, J). This positioning of Pacsin3 at the cell surface is very reminiscent of the subcellular localization of Caveolin-3-GFP [36] and of the planar cell polarity protein prickle and ERM proteins on the plasma membrane of notochord cells in the primitive chordate *Ciona intestinalis* [37].

Dendrograms of the PACSIN protein family clearly show that zgc:56324 is the only member of the PACSIN 3 branch in *D. rerio* (Figure 3A). This Pacsin isoform is overall 61% identical to the *Xenopus laevis* PACSIN 3 and 62% identical to human PACSIN 3. The EFC and SH3 domains of the zebrafish Pacsin3 are 44% and 64% identical to *Drosophila* Syndapin, respectively. Yet, unlike the PACSIN 1 and PACSIN 2 orthologues, zebrafish Pacsin3 has a shortened unstructured linker region between the EFC and SH3 domain that lacks NPF triplets, which allow binding to EH domain proteins [38] (Figure 3I). Analogous to the mammalian PACSIN 3 [21], [33] and *Xenopus* counterparts, neither the pufferfish *Fugu rubripes* nor *Tetraodon nigroviridis* Pacsin3 orthologues have NPFs, and translated ESTs from other fish (*Salmo salar* (salmon), DW581863; *Onchorhynchus mykiss* (trout), CA386865; and *Pimephales promelas* (minnow), DT364755) also do not contain NPF motifs within the linker polypeptide bridging the EFC and SH3 domains. Pacsin3 may thus only interact with a subset of the endocytic machinery.

**Figure 4:**
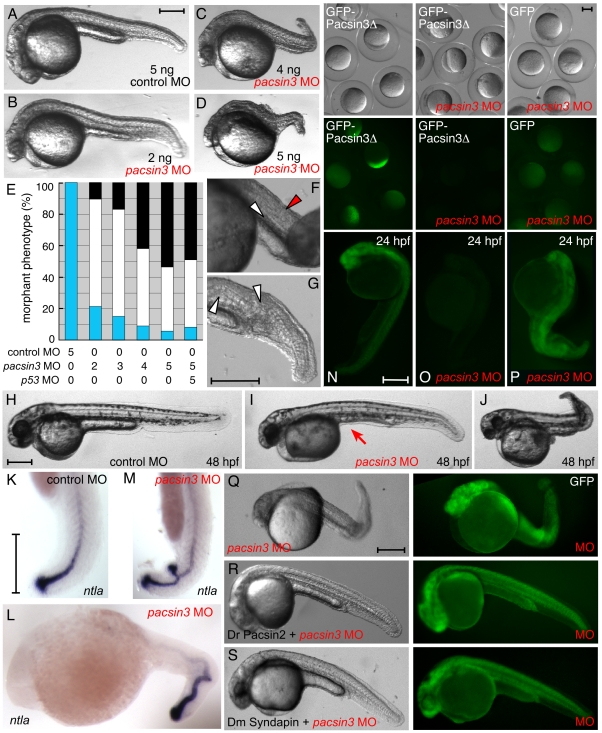
Inactivation of Pacsin 3 in zebrafish embryos. (A–D) Morphology of representative control (scrambled) or *pacsin3* MO-injected 24 hpf embryos. Anterior is left. Bar  = 250 µm. (E) Phenotypic quantitation of normal (blue), mild (white), or severely (black) affected embryos injected with control (*n* = 42) or 2 ng (*n* = 48), 3 ng (*n* = 53), 4 ng (*n* = 57), or 5 ng (*n* = 54) *pacsin3* MO, or with both 5 ng *pacsin3* and 5 ng *p53* MOs (*n* = 61). (F–G) Close-up views of abnormal notochord (white arrowheads) and improperly structured somites (red arrowhead) in 24 hpf *pacsin3* MO-injected embryos. Bar  = 250 µm. (H–J) Representative lateral views of control or *pacsin3* MO-injected 48 hpf embryos. Note lack of the yolk tube (arrow) even in mildly affected *pacsin3* morphants. Bar  = 250 µm. (K–M) Localization of *ntla* mRNA in typical control or *pacsin3* MO-injected 24 hpf embryos. Anterior is up in K and M. Bar  = 250 µm. (N–P) GFP fluorescence from injection of 25 pg GFP-Pacsin3Δ (N, O) or GFP (P) mRNA into embryos together with no (N) or 5 ng *pacsin3* MO (O, P) at the one-cell stage. Groups of embryos still within the chorion at ∼5 hpf and typical individual 24 hpf embryos show effective and selective silencing of the *pacsin3* transcript. Bar  = 250 µm. (Q–S) Gross morphology of 3 ng *pacsin3* MO-injected embryos co-injected with 50 pg GFP (Q) or 50 pg GFP and either 25 pg *D. rerio* Pacsin2 (R) or *D. melanogaster* Syndapin (S) mRNA. Bar  = 250 µm.

The highly localized expression of Pacsin3 during early embryogenesis suggested the possibility of silencing the transcript as a means to address structure–function relationships. Injection of *pacsin3* AUG antisense morpholino oligonucleotide (MO) (Table S2) into wild-type one- or two-cell embryos causes severe developmental abnormalities, in a dose-dependent manner (Figure 4A–E and S4). At 24 hpf, severely affected embryos are grossly malformed with poorly differentiated notochords, shortened posteriors, kinked body axes and distorted somites compared with control embryos (Figure 4F, G). The most severely affected embryos lack a yolk tube extension (Figure 4C, D), which forms during the segmentation period along with the notochord [39]. In moderate-to-severely affected embryos at 48 hpf, the pronounced body axis abnormalities (Figure 4H–J) lead to uncoordinated twitching or gyration when touched, while larvae injected with control scrambled MO rapidly advance forward linearly [40]. Embryos injected with 10 ng MO do not survive to 24 hpf.

The *no tail a* (*ntla*, zebrafish *Brachury*) transcription factor is required for notochord development and tail formation [41] and, in one-day old wild-type embryos, the transcript is highly expressed in the tailbud and at a lower level in the rod-like notochord (Figure 4K). In *pacsin3* MO-injected embryos, the *ntla* message reveals the presence of an undulating/spiral notochord (Figure 4L). *ntla* expression indicates that specification of notochord fate is not defective and rather suggests later notochord development abnormalities reminiscent of *crash test dummy, zickzack* or *wavy tail* mutants [41]. The undulation is also comparable to the *sneezy* and *happy* mutant phenotype [42], [43]. In some severe cases after Pacsin3 depletion, in addition to the kinked notochord, aberrant development of two tailbuds is evident (Figure 4M).

Coinjection of a *p53*-silencing MO with the *pacsin3* MO does not significantly alter the phenotypic outcome (Fig. 4E), indicating that knockdown-associated p53 activation does not exacerbate the phenotype [44]. In addition, the expression in embryos of an ectopic, fluorescently-tagged Pacsin3 lacking the SH3 domain (Pacsin3Δ) (Figure 4N) is effectively extinguished by *pacsin3* MO, as early as the shield stage of gastrulation and up to 24 hpf (Figure 4O). By contrast, coinjection of GFP with the MO has no effect on the GFP fluorescence, as expected (Figure 4P) [45].

### Pacsin3 and Notochord Differentiation

The notochord is a defining feature of all chordate embryos, and is involved in morphogenesis and body patterning during development [46]. A mesoderm-derived structure situated between the neural tube and forming internal organs, this is a consequence of the two major functions of the notochord: First, it acts as a midline axial structure that provides physical support before the appearance of the bony skeleton in higher vertebrates. Second, the notochord produces and secretes diffusible morphogens, like Sonic hedgehog, which guide the placement and differentiation of adjacent body structures and organs. Notochord contains a single cell type that secretes a dense extracellular matrix to encapsulate the whole rod-like structure [46]. After assembly of the overlying, laminin- and collagen-rich perinotochordal basement membrane sheath, the individual enclosed notochord cells, in an anterior to posterior sequence, vacuolate internally. Fluid-filled vacuoles ultimately occupy up to 80% of the cell volume. The hydrostatic pressure of vacuolated notochord cells against the taut overlying sheath generates the mechanical properties of the notochord in early embryogenesis. An undulating notochord between 12 and 24 hpf can be a consequence of defects in either sheath assembly or vacuolation. For example, *sneezy, happy* and *dopey* are all components of the COPI complex that operates along the secretory pathway and mutants display major defects in extracellular matrix exocytosis [43]. Similarly, the *sleepy* and *grumpy* loci encode laminin chains which, when defective, cause aberrant assembly of the trilaminar sheath [47]. Chemical inhibitors of, or genetic errors in, lysyl oxidases that crosslink the basement membrane [48], [49], or in the α1 chain of type VIII collagen [50] likewise prevent proper formation of the notochord sheath.

**Figure 5:**
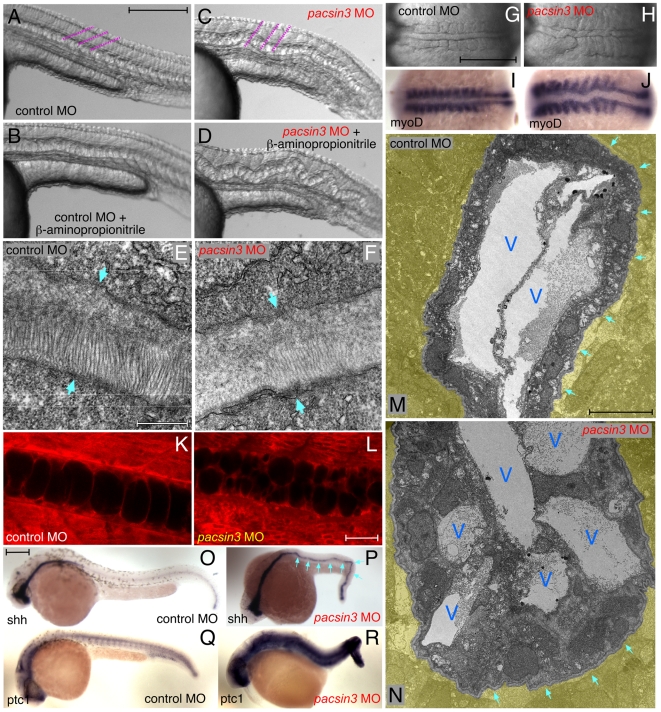
The *pacsin3* MO phenotype. (A–D) Lateral notochord morphology in 5 ng control or *pacsin3* MO-injected 24 hpf embryos treated with (B and D) or without (A and C) 10 mM β-aminoproprionitrile to disrupt the notochordal sheath. The relative angle of the normally chevron-shaped somites is indicated (purple). Anterior is left. Bar  = 250 µm. (E–F) Thin section EM images of the trilaminar perinotochordal sheath in 5 ng control or *pacsin3* MO-injected 24 hpf embryos. Arrows demarcate the boundary of the sheath. Bar  = 0.5 µm. (G–J) Close-up dorsal views of the chordamesoderm at the 10-somite stage in 5 ng control (G and I) or *pacsin3* (H and J) MO-injected embryos. Anterior is left. (I–J) Embryonic *myoD* mRNA localization by whole mount *in situ*. Bar  = 250 µm. (K–L) Representative confocal sections of the lateral notochord region from BODIPY-Texas red labeled [88] live 24 hpf embryos after 5 ng control or *pacsin3* MO injection. Bar  = 50 µm. (M–N) Thin section EM micrographs of cross-sections through the notochord of 5 ng control or *pacsin3* MO-injected 24 hpf embryos. Extra-notochord tissue is pseudocolored yellow, and vacuoles (V) and the perinotochordal sheath (arrows) are indicated. Bar  = 10 µm. (O–R) Embryonic *sonic hedgehog* (*shh*) and *patched1* (*ptc1*) mRNA localization by whole mount *in situ* in 5 ng control or *pacsin3* MO-injected 24 hpf embryos. Bar  = 250 µm.

Treating control MO-injected embryos with β-aminoproprionitrile, a lysyl oxidase inhibitor, causes a wavy notochord at 24 hpf (Figure 5A, B) [49]. Similar treatment of *pacsin3* MO-injected embryos results in remarkable accordion-like pleating of the notochord (Figure 5C,D). This exacerbated phenotype suggests that compromising the pericellular sheath worsens the effect of Pacsin3 depletion and that Pacsin3 may not play a direct role in exocytosis and assembly of the sheath. Indeed, electron microscope (EM) analysis shows the typical trilaminar arrangement of the basal lamina in both control and *pacsin3* morphants (Figure 5E, F). Certain toxicants induce a very similar undulating notochord sheath defect [51]–[53], which can be completely suppressed by inhibiting spontaneous myotome contractions that commence around 17 hpf [54]. Yet, in *pacsin3*-morphant embryos, a misshapen notochord is already visible at the 6–10-somite stages (12–14 hpf) (Figure 5G, H), arguing against the defect being directly related to sheath abnormalities or contraction-induced distortions. Instead, the Pacsin3-deficient embryos appear to have a differentiation and vacuolation defect. The notochord is broader, twisted and undulating (Figure 5H, J), grossly mirroring the defect associated with *pipetail* mutations at gastrulation [55]. Adaxial expression of *myoD* transcripts, encoding a transcription factor involved in muscle differentiation [56], is clearly abnormal in *pacsin3* morphants. Frequently, somites are expanded laterally adjacent to the wider, meandering notochord in *pacsin3* morphants, also indicative of defective segmentation (Figure 5J). At 24 hpf, notochord cells contain more numerous, only partially expanded vacuoles (Figure 5K, L); the notochord cell length-to-width ratio decreases from the control 2.07 to 1.04 (*n* = 98) in *pacsin3* morphants, and EM shows smaller intracellular vacuoles and increased number of undifferentiated cells within the notochord (Figure 5M, N). Thus, at 24 hpf, rounded cells have clustered at the dorsal midline but fail to properly intercalate and expand. Compared with 24 hpf controls, in MO-injected embryos transcripts for the early marker genes *sonic hedgehog* in the notochord [57], and *patched* in surrounding target tissue, are persistently elevated (Figure 5O–R) [58], further suggesting a differentiation defect [43], [47], [59], [60].

### Cell Locomotion Defects in *Pacsin3* Morphants

**Figure 6:**
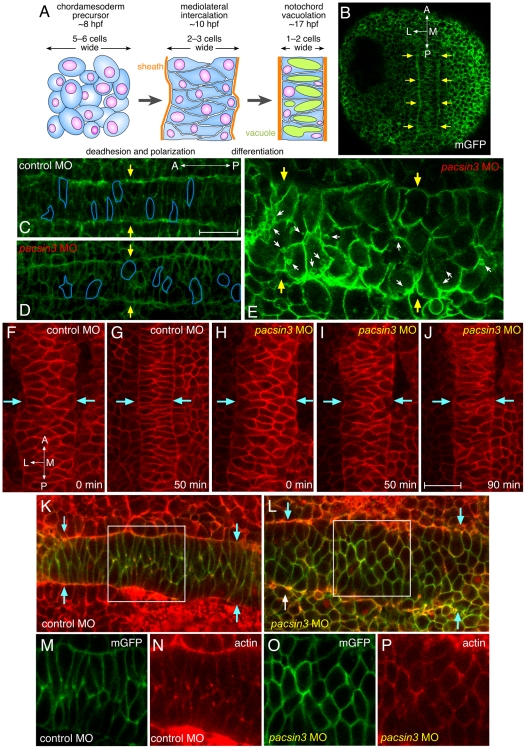
Early midline defects in *pacsin3* morphants. (A) Schematic illustration of the mediolateral intercalation process in the forming notochord. (B–C) Representative confocal optical sections of fixed, mGFP and control MO-injected embryos at the 3-somite stage focused on the mesodermal cell layer. The lateral notochord border is indicated (arrows), as are the anterioposterior and mediolateral axes. Bar  = 50 µm. (D–E) Representative confocal optical sections of fixed, mGFP and 5 ng *pacsin3* MO-injected embryos at the 3-somite stage with moderate (D) or severe (E) phenotypes. The shape of several cells is traced (blue lines), and internal membrane vesicles (small arrows) are shown. (F–J) Selected dorsal midline views from time-lapse recording at the beginning of segmentation (∼10 hpf) of control (7.5 ng; F, G and Video S1) or *pacsin3* (7.5 ng; H–J and Video S2 and S3) MO-injected embryos also expressing membrane mcRFP. The lateral notochord border is indicated (arrows). Bar  = 50 µm. (K–P) Representative confocal optical sections of fixed, mGFP and control or *pacsin3* MO-injected embryos at the 3-somite stage stained with fluorescent phalloidin to reveal actin. Anterior is left. The lateral notochord border is indicated (K and L, arrows), and the separated mGFP (green) and actin (phalloidin, red) channels of the regions boxed in K and L are shown (panels M and O and N and P, respectively).

In zebrafish embryos, the notochord rudiment is initially a field ∼20 cells wide that, over the course of ∼8 hours, converge into parallel-arrayed column [61] (Figure 6A). These morphogenetic changes that underpin notochord formation can be visualized with membrane-tethered GFP (mGFP) [62]. At the 3-somite stage, a clear parallel boundary between the midline notochord and adjacent adaxial mesoderm is evident (Figure 6B, C). The notochordal cells are polarized in a mediolateral direction as they undergo intercalation to form a single column of cells. By contrast, in severely affected *pacsin3* morphants, the adaxial border is jumbled and the prenotochordal cells are rounded and not properly oriented mediolaterally (Figure 6D, E). Comparable time-resolved images of control and *pacsin3* MO-injected embryos reveal that at the onset of segmentation (∼10 hpf), Pacsin3-deficient chordamesoderm fails to intercalate properly (Figure 6F–J and Video S1, S2, S3, S4). Although the width of the notochord decreases, this appears largely due to compression by the adjacent lateral cells undergoing active convergence and extension (Video S1, S2, S3, S4). The rounded shape of many cells does not parallel that of control notochord and in *pacsin3* morphants, the notochord undulates out of the plane of focus. In comparison, 30 min after the control embryos have aligned into a two-cell-wide column oriented along the anterioposterior axis [63], Pacsin3-depeleted embryos still have not intercalated correctly (Video S3 and S4). This strongly suggests defective cellular migration during intercalation.

Abnormalities in the cell shape changes that accompany medial-directed migration are also seen in fixed, 3-somite stage *pacsin3* MO embryos stained with fluorescently-labeled phalloidin. In contrast to the mediolaterally-elongated cells in control embryos, with actin staining concentrated at the sites of cell–cell intercalation, the *pacsin3* morphant chordamesoderm is populated with numerous rounded cells exhibiting circumferential cortical actin (Figure 6K and L); Considerably less evidence of the focally polarized actin at mediolaterally-oriented cell contacts seen in control notochord is apparent in the abnormally-forming notochord of *pacsin3* MO-injected embryos (Figure 6M–P).

Altogether, we conclude that Pacsin3 is involved in notochord differentiation, and that the reproducible body patterning abnormalities that follow *pacsin3* MO injection are due to depletion of Pacsin3. Developmental failure of the notochord to extend and rigidify can account for the shortened posterior and distorted/malformed trunk and tail. Strong confirmation of this action of Pacsin comes from the ability to grossly restore normal development in the vast majority of MO-treated embryos by coinjection of capped RNA encoding either zebrafish Pacsin2 (77.3% normal; *n* = 128) or *Drosophila* Syndapin (84.8%; *n* = 185) (Figure 4Q–S). At the concentration of injected mRNA, these full-length proteins alone do not display any dominant gain-of-function effects (Figure S5). The lack of a full-length cDNA precluded reconstitution experiments with the *Danio* Pacsin3 isoform.

### Structure–Function Analysis

**Figure 7:**
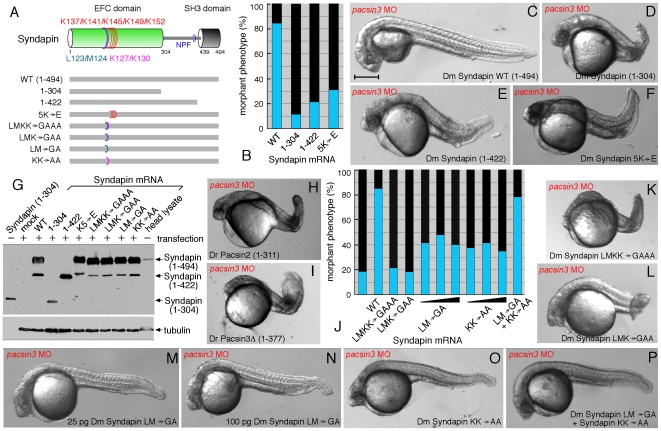
Structural requirements for Syndapin operation. (A) Schematic depiction of the various Syndapin mutants analyzed. (B) Quantitative phenotypic analysis of normal (blue) or abnormal (black) *pacsin3* (3 ng MO) morphants coinjected with 25 pg Syndapin WT (*n* = 185) or Syndapin 1–305 (*n* = 97), 1–423 (*n* = 98) or 5K→E (*n* = 121) mutant mRNAs. Bar  = 250 µm. (C–F) Representative images of the phenotype of 24 hpf *pacsin3* MO embryos expressing the indicated Syndapin proteins. (G) Immunoblot analysis of expression of various Syndapin fragments and mutants from transfected pCS2+ in HeLa cells. The anti-Syndapin serum recognizes the EFC domain; as positive controls, purified Syndapin (1–304) and a *Drosophila* head lysate containing endogenous Syndapin were used. (H–I) Representative images of *pacsin3* MO embryos also expressing *D. rerio* Pacsin2 or Pacsin3 SH3Δ proteins. (J) Quantitative phenotypic analysis of normal (blue) or abnormal (black) *pacsin3* (3 ng MO) morphants alone (*n* = 50) or coinjected with 25 pg Syndapin WT (*n* = 185), LMKK→GAAA (*n* = 119), LMK→GAA (*n* = 99), LM→GA (*n* = 95; 50 pg, *n* = 48; 100 pg, *n* = 43), KK→AA (*n* = 122; 50 pg, *n* = 57; 100 pg, *n* = 76) or 25 pg of both the LM→GA and KK→AA (*n* = 115) mutant Syndapin mRNAs. (K–P) Representative images of the phenotype of *pacsin3* MO embryos expressing the indicated Syndapin proteins.

Because the ectopic *Drosophila* Syndapin RNA could rescue the development of *pacsin3* MO-injected embryos with high frequency, we utilized this morphologic complementation assay to assess the functional significance of the PACSIN/Syndapin EFC and SH3 domains (Figure 7A–F). Coinjection of mRNA encoding just the Syndapin EFC domain (residues 1–304) is unable to counteract the loss of Pacsin3. In addition, the range of pronounced flexed body axis phenotypes caused by injection of 3 ng MO together with 25 pg SyndapinΔ (residues 1–422), or the full-length Syndapin EFC domain 5K→E mutant (which abrogates liposome binding *in vitro*) encoding RNA are essentially indistinguishable from those seen in embryos injected with the MO alone (Figure 7B, E, F). Importantly, failure to ameliorate the consequences of Pacsin3 loss is not due to lack of protein expression since antibodies against Syndapin confirm synthesis of all the mutant proteins analyzed from the transfected pCS2+ plasmid (Figure 7G). Removal of the SH3 domain from either *Danio* Pacsin2 or -3 also abolishes the ability of these proteins to restore normal development in *pacsin3* MO-injected embryos (Figure 7H, I). These results therefore reveal that the EFC and SH3 domains must be both functional and physically connected for Syndapin to compensate for loss of Pacsin3 in zebrafish embryos.

We next addressed the functional role of the protruding loop region upon the concave surface of the EFC domain identified in the structure. Introducing the compound LMKK→GAAA mutation into the prong region completely abrogates the ability of the expressed mutant protein to overcome the deleterious effects of *pacsin3* MO injection. A triple LMK→GAA substitution is similarly inactive (Figure 7J–L). Interestingly, the LM→GA mutation partially rescues the *pacsin3* MO-coinjected embryos, suggesting that Syndapin harboring this mutation has some activity (Figure 7M). The incidence of apparently normal embryos at 24 hpf is roughly double that of the related triple and quadruple mutant-injected embryos, but still only about half that obtained from coinjection of the wild-type Syndapin RNA (Figure 7). The paired LM→GA substitution does not seem to incapacitate Syndapin due to a reduced apparent affinity, as there is little dose-dependent effect of mRNA injection (Figure 7N). We interpret this to indicate that the region that encodes the hallmark Syndapin/PACSIN insert within the membrane-apposed face of the EFC domain (Figure 2) is necessary for the optimal and correct functioning of these proteins. Supporting this idea is the severely diminished rescuing capacity (72% morphologically abnormal, *n* = 95) of the zebrafish Pacsin2 harboring an IIKK→GAAA mutation within the slightly longer prong loop typical of the vertebrate orthologues (not shown) [28]. Simultaneous mutation of K127 and K130 in Syndapin also results in partial rescue, giving an intermediately active form yielding roughly double the number of normal-appearing embryos at 24 hpf than injection of either the LMKK→GAAA or LMK→GGA along with the *pacsin3* MO (Figure 7J, O). Again, using different injected mRNA concentrations, the modest activity of the mutant Syndapin suggests little evidence of a dose-dependent effect for the KK→AA mutant.

To better understand any integrated role for the prong aliphatic side chains (L123, M124) along with the adjacent basic residues (K127, K130) in Syndapin operation, we assayed for *trans* complementation by injecting mRNA encoding either pair of mutations alone or both transcripts together. While neither mutation alone can fully reverse the effect of *pacsin3* MO-mediated gene knockdown, coinjecting the two mutants together promotes outwardly normal development at 24 hpf (Figure 7J, P). Because both mutant proteins are expressed (Figure 7G) and likely coassemble to form antiparallel dimers, the results suggest that only a single intact prong surface is necessary for Syndapin activity in the context of the assembled dimer, and so oligomerization is an integral part of PACSIN function. The data also argue for the exposed hydrophobic loop residues and vicinal basic side chains performing physically separable functions, but the loss of the lysines in the reciprocal dimer partner could be compensated by the intact opposite chain and/or by other functionally important basic residues, as typified by the 5K→E mutant. Together, these functional studies show the protrusive hydrophobic prong characteristic of the Syndapin/PACSIN protein EFC domain is an integral functional surface that contributes to the proper activity of the whole protein; our data confirm and extend considerably *in vivo* the recent structural work on the PACSIN 1/2 F-BAR domains [28].

## Discussion

The participation of the secretory pathway in notochord formation is well established in zebrafish; extracellular matrix biosynthesis and constitutive exocytosis are essential for correct assembly of the perinotochordal sheath [43], [46], [47]. Yet, the role of endocytic components in stereotyped cell movements during notochord mediolateral intercalation is poorly understood. Our data suggest plasma membrane dynamics and the endocytic pathway play an equally important role in the proper development of the notochord. The endocytic protein PACSIN/Syndapin binds with high affinity to PtdIns(4.5)P_2_, a lipid localized overwhelmingly to the plasma membrane [64]. At steady state, Pacsin3 is positioned at the surface of parallel-arrayed notochord cells. Importantly, the tissue defect resulting from extinguishing Pacsin3 is quite unlike that caused by pharmacologic inhibition of lysyl oxidases, where notochord differentiation (vacuolation) is normal [48]. This means that the failure to differentiate properly is not due to aberrant basement membrane assembly or organization.

*Drosophila Syndapin* loss of function is semi-lethal [65]. In the larval nervous system, Syndapin appears to be positioned postsynaptically and not participate directly in synaptic vesicle exo/endocytosis at the presynaptic plasma membrane [29]. Forced expression in muscle causes massive expansion of a tubular subsynaptic reticulum at the neuromuscular junction, validating the strong membrane tubulation activity of Syndapin [65]. Strikingly though, subsynaptic reticulum morphology does not change obviously in *synd* homozygous mutant larvae and, because no synaptic transmission deficits are associated with the gain-of-function phenotype, *in vivo* analysis is restricted to an overexpression-induced morphological aberration [65]. By contrast, in zebrafish, *pacsin3* silencing leads to a penetrant, severe developmental phenotype consistent with a primary failure in notochord differentiation. The earliest defect we detect in *pacsin3* morphants appears to be a breakdown in mediolateral intercalation behavior at the end of gastrulation and the onset of segmentation. This argues that Pacsin3 participates in the regulation of cell migration in a key manner. While the precise molecular basis for the abnormal intercalation remains to be comprehensively defined, we believe it is likely to reflect faulty cellular locomotion as a consequence of endocytic abnormalities. Continual disruption and reestablishment of both cell-cell and cell-matrix attachments is necessary for the coordinated movement toward the dorsal midline [66]. Endocytosis of integrin, cadherin and/or other membrane-embedded receptors is likely required to remodel the plasma membrane during these movements to allow presumptive notochord cells to align single file in a column oriented along the anterioposterior axis [63]. For example, deadhesion of notochord cells requires uptake of the EphrinB1 receptor in a dynamin-dependent fashion [63]. Polarization along the mediolateral axis requires that protrusive activity and traction does not occur productively at the anterioposterior or dorsovental surfaces of the chordamesoderm during intercalation. Endocytosis may thus play a key role in defining embryonic axes to facilitate appropriate transverse convergence. Supporting a general role for Pacsin3 in receptor-mediated endocytosis during the gastrula period and segmentation in the zebrafish is the similar prominent undulating notochord at 14 hpf in embryos injected with dynamin (*Dnm1*) MO or ectopically expressing a dominant-negative form of dynamin [63]. We propose that in Pacsin3 MO tissue, endocytic insufficiency leads to failed polarization of the notochord along the mediolateral axis and concomitant aberrant cell migration and differentiation patterns.

Pacsin3 depletion impacts only a subset of developmental movements. The three germ layers still form properly by involution, and convergence and extension of the lateral mesoderm is still intact. While this could mean that Pacsin has a selective endocytic activity only within prenotochordal cells, we believe that a specific role in notochord maturation is due to the highly restricted Pacsin3 mRNA expression pattern. In *Xenopus laevis*, PACSIN2 is ubiquitously expressed in the developing embryo [67]. Ectopic PACSIN2 expression interferes with integrin α5β1 activation and clustering at focal adhesions and therefore appears to disrupt gastrulation by disturbing proper cell migration [67]. Pacsin3 may operate similarly but this isoform is highly localized to the notochord in zebrafish embryos and clearly not involved generally in cell movements during gastrulation. Moreover, that the ∼50% identical *Drosophila* Syndapin can rescue the *pacsin3*-morphant phenotype indicates that the homologous gene products from vertebrates and invertebrates perform analogous molecular functions [57]. Diversification of the PACSIN gene family in chordates (Figure 3A) likely allows differential tissue expression patterns for the paralogs. This is indeed the case for Pacsin2 and Pacsin3 in the zebrafish and, in humans (and mice), PACSIN 1 is expressed chiefly in the nervous system [33], [68].

Defective notochord vacuolation in *pacsin3* morphants may simply be a secondary consequence of failed intercalation and differentiation. However, we suspect this might reflect a second, later activity of Pacsin3. Transverse movements of the forming notochord are completed at ∼14 hpf [61] yet the zygotic *pacsin3* transcript and protein is strongly expressed for at least another 24 h. This is when vacuolation occurs, beginning around 15 hpf and reaching completion ∼36 hpf [48]. In mammals, PACSIN 3 associates with the osmotic and mechanosensitive vanilloid-type transient receptor potential channel TRPV4, regulating both subcellular positioning and basal Ca^2+^ channel activity [69], [70]. Remarkably, zebrafish Trpv4 is expressed highly selectively within the notochord along with Pacsin3 during segmentation [71] and, suggestively, *trpv4* morphants have a shortened, twisted body axis [72]. Future experiments will explore any role for similar interactions in zebrafish.

Using a gross morphological zebrafish embryonic development assay, we find that the combinatorial use of the PACSIN high affinity PtdIns(4,5)P_2_-binding EFC domain and the dynamin/WASp-binding SH3 domain is essential for the normal operation of these proteins [19], [28], [65]. Our functional studies also reveal unambiguously that the prong our structural data show is unique to the Syndapin/PACSIN EFC domain is necessary for the activity of both invertebrate and vertebrate proteins. Why PACSIN EFC domains have a smaller radius of curvature and an obligate prong may be related to the synaptic requirement for PACSIN 1 in vertebrates only after persistent stimulation at high frequency [19], [73]. This entails clathrin-mediated endocytic uptake from large internalized membrane vesicles [19], [73]. We in fact observe obvious internal membrane intermediates tagged with mGFP in abnormally migrating notochordal cells from *pacsin3* morphants (Figure 6E). Bulk endocytosis of substantial portions of surface membrane coupled with Pacsin-dependent budding may possibly be required in intercalating cells for polarized elongation or motility. The prongs and geometry of the PACSIN EFC dimer could also be related to coupling with actin. Actually, it is proposed that the bulk endocytic uptake phenotype and actin abnormalities are functionally interconnected [19]. Alternatively, cellular locomotion depends heavily on focal actin nucleation and extension, and the protrusive force that drives cell polarization and traction in intercalating notochord requires cytoskeletal rearrangements (Skoglund et al., 2008), and may be impacted by Pacsin3. In *X. laevis*, notochord cells can be isolated from embryos and cultured from explants [74], [75]. While our current work provides a functional framework for the participation of PACSIN in tissue development, in the future, this type of single cell-based analysis coupled with MO-mediated translational silencing could provide greater mechanistic insight into PACSIN operation during notochord formation.

## Materials and Methods

### DNA and RNA Procedures

The *Drosophila Syndapin* cDNA clone was obtained from the Drosophila Genomics Resource Center and cloned into pGEX-4T-1 and pCS2+. The zebrafish Pacsin2 cDNA and a Pacsin3 partial EST clone truncated at residues 377 were obtained from Open Biosystems and both cloned into multiple cloning site I in pCS2+ using PCR. The zebrafish Pacsin3 (1–377) PCR fragment was also cloned into pGEX-4T-1. Syndapin (1–305) and (1–423) and zebrafish Pacsin2 (1–311) truncation mutants were constructed using QuikChange mutagenesis (Stratagene) to convert appropriate base pairs to stop codons. All point mutations within the *Drosophila* Syndapin, ^123^LM KK→GAAA,^ 123^LMK→GAA,^ 123^LM→GA, ^127^KK→AA, and the zebrafish Pacsin2 ^122^IIKK→GAAA in were generated by QuikChange mutagenesis. All constructs were verified by automated dideoxynucleotide sequencing and full details of the mutagenic primers are available upon request.

The *ptc1* clone in pBluscript (KS) was a gift from Alexander Schier (Harvard University) while the mGFP [62] and mcRFP plasmids were from Lilianna Solnica-Krezel (Vanderbilt University). cRNA was synthesized from pCS2+ clones linearized by digestion with NotI, using mMESSAGE mMACHINE SP6 kit (Ambion) according to the manufacturers protocol.

### Protein Expression and Purification

Full-length Syndapin was expressed in BL21 (DE3) pLysS cells using a standard induction protocol [76]. For selenomethionine-substituted Syndapin, a colony was grown for ∼8 hours at 37°C in 2x TY containing ampicillin (100 µg/ml) and chloramphenicol (50 µg/ml) after which 200 µl was diluted in 200 ml of minimal media and grown overnight at 37°C. 10 ml of this overnight culture was added to 1 l of minimal media and grown at 37°C until an OD_600_ ∼0.6, when 0.5 g of an amino acid mixture (1.2 g each of lysine, threonine and phenylalanine; 0.6 g each of leucine, isoleucine and valine) and 0.6 g of L(+) selenomethionine (ACROS Organics) was added. Approximately 15 min later, the cells were induced with 0.2 mM IPTG and the temperature shifted to 25°C for overnight growth. Cells were pelleted and resuspended in 10 mM HEPES-OH, pH 7.5, 200 mM NaCl (buffer A) containing 5 mM DTT, 4 mM MnCl_2_, DNAse and the protease inhibitors AEBSF and benzamidine. Following mechanical lysis, the insoluble material was sedimented, the soluble fraction incubated with glutathione-Sepharose and cleaved Syndapin eluted off the column following overnight thrombin treatment. Syndapin was concentrated and loaded onto a Superdex 200 gel filtration column equilibrated in buffer A with 5 mM DTT. Syndapin containing fractions were pooled, concentrated and screened for crystallization conditions using commercially available sparse matrix crystallization screens from Hampton Research and Molecular Dimensions.

### Crystallization, Data Collection and Structure Determination

Crystals grew as plates from 17% PEG 3350, 0.1 M disodium phosphate in hanging drop trays. Crystals were cryoprotected in a stepwise process such that 0.5 µl aliquots of cryoprotection buffer (25% glycerol, 0.15 M salt, 18% PEG 3350) was added to a 3 µl drop of stabilizing solution containing a crystal until a final drop volume of ∼10 µl (glycerol concentration ∼17.5%). The crystal was then transferred into two successive drops of 100% cryoprotection buffer, flash-frozen in liquid nitrogen and later mounted.

Wavelength scan of these crystals on beamline ID29 at the European Synchrotron Radiation Facility showed the Peak  = 12.6620 keV, inflection 12.6601 keV and remote at 12.7080 keV. Data was collected at the peak wavelength, indexed with Mosflm [77] and scaled with SCALA [78], [79]. Six of an expected eight selenomethionine sites were found using autoSHARP [80] followed by phasing the density modification in SHARP [81]. The resulting solvent flattened map, with 60.5% solvent content and two molecules in the asymmetric unit, showed obvious helical structure that was readily modeled using the ‘place helix here’ option in Coot [82]. Several rounds of model building and refinement in REFMAC5 [83] produced the final model which contains residues 14–303 of one monomer (chain A) and residues 14–298 of the other (chain B), 104 water molecules (chain W), two glycerol molecules (chain C) and three sodium ions (chain D). Residues 1–13 of each monomer and residues 169–188 of chain A are missing due to poor electron density. Data collection and refinement statistics are reported in Table S1. The coordinates of the Syndapin EFC domain have been deposited with the PDB accession code 3I2W.

### Liposome Preparation and Binding Assay

Liposomes composed of 10% cholesterol, 35% PtdCho, 35% PtdEth, 10% PtdSer and 10% of either PtdIns or PtdIns(4,5)P_2_ were prepared and used in sedimentation assays precisely as described [84]. PtdSer liposomes contained 30% PtdSer and no PtdIns or PtdIns(4,5)P_2_. For negative stain EM, 3–4 µl of protein (0.15 mg/ml) incubated with Folch brain liposomes (0.2 mg/ml) on ice for 2–5 min was immobilized on a formvar coated grid for 7 min, the excess sample wicked off and the grid stained with 3 µl of uranyl acetate for 1–3 min. After removal of excess stain, the sample was imaged on a Jeol 1011CX transmission EM.

### Surface Plasmon Resonance

All lipids (synthetic) were from Avanti Polar Lipids (USA). The preparation of liposomes and their capture to the L1 surface of a BIAcore 3000 biosensor was exactly as described [32]. All proteins were tested at concentrations ranging from 25 nM to 2 µM for binding to liposomes (70% PtdCho, 20% PtdEth and 10% PtdIns(4,5)P_2_) in 10 mM HEPES, pH 7.4, 100 mM NaCl or 10 mM Tris, pH 8.7, 250 mM NaCl (AP-2). Binding to liposomes without PtdIns(4,5)P_2_ served as a negative control and was subtracted from the original sensorgrams prior to evaluation. The rate constants were calculated with the evaluation software (version 4.1, provided by the manufacturer) and assuming a 1∶1 mode of binding.

### Antibodies

Pacsin3 (1–377) cleaved from GST with thrombin was injected into rabbits (PickCell Laboratories). Anti-Pacsin3 antibodies were affinity purified from immune serum using thrombin-cleaved Pacsin3 (1–377) coupled to CNBr-activated Sepharose after preadsorbing the serum with immobilized GST-DnaK to remove cross-reacting anti-Hsp70 antibodies. Guinea pig anti-Syndapin serum was kindly provided by Vimlesh Kumar (University of Dublin Trinity College) and the anti-tubulin mAb E7 was from the DSHB. Alexa488-conjugated anti-rabbit IgG was purchased from Invitrogen.

### Zebrafish Maintenance, RNA Injections, and In Situ Hybridization

The Oregon AB* strain was maintained under standard conditions at the University of Pittsburgh School of Medicine in accordance with Institutional and Federal guidelines for use, care and maintenance of experimental animal models and with University of Pittsburgh Institutional Animal Care and Use Committee (IACUC) approval. Embryos from natural matings were obtained and developmentally staged [39]. The injection procedures were performed as described previously [85] with the following modifications for RNA injections: wild-type zebrafish embryos were injected with 25–100 pg *Syndapin*, *pacsin2* or *pacsin3Δ* mRNA at the one- to two-cell stage. For cell membrane labeling, 50–100 pg mGFP or mcRFP mRNA was similarly injected. All injected embryos were incubated in E3 medium (5 mM NaCl, 0.17 mM KCl, 0.33 mM CaCl_2_, 0.33 mM MgSO_4_, 0.01% methelene blue) at 28°C. For the β-aminoproprionitrile experiments, from 3 hpf on, embryos were incubated in 15 mM HEPES-OH, pH 7.6 buffered E3 supplemented with 10 mM β-aminoproprionitrile (Sigma) at 28°C until microscopic analysis.

For whole mount *in situ* analysis, antisense oligonucleotide probes were synthesized for *pacsin3*, *ntla*, *myoD*, *shh*, and *ptc1*. Hybridization riboprobes were made from full-length or partial clones in pBluscript (KS). Typically, about 1 µg plasmid was linearized and a digoxigenin-labeled RNA probe was synthesized by T7 RNA polymerase (Roche) according to the manufacturer's procedures. Appropriate zebrafish embryos were fixed in 4% paraformaldehyde at 4°C overnight, washed once in PBS and stored in 100% methanol at −20°C. Whole-mount *in situ* hybridization was done by standard protocols [86], [87]. For microscopy, dechorionated embryos were mounted in 3% methylcellulose and overlaid with E3 containing 0.016% tricaine (pH 7.0). Oriented embryos were viewed with a Leica MZ16FA stereo fluorescence microscope with a 1x (NA 0.14) objective and images captured with a QImaging Retiga-EXi Fast 1394 digital camera. TIFF images were cropped in Adobe Photoshop and assembled with Macromedia Freehand or Adobe Illustrator.

### Antisense MO Injections

A complementary MO (5′-ATCCGTCCATGTCACCTGGGTCTTC-3′) targeting the initiation codon of *pacsin3* (Table S2) was designed by GeneTools. The *pacsin3* or control (5′-CCTCTTACCTCAGTTACAATTTATA-3′) MO was injected into the yolk of one- to two-cell stage embryos incubated in E3 at 28°C until the desired stage for fixation and *in situ* studies. To determine the morpholino specificity, one-cell stage embryos were injected with an EGFP fusion construct (50 pg) consisting of the 5′UTR and amino acids 1–377 of *pacsin3* fused to the coding sequence of EGFP. These same embryos were injected with control or *pacsin3* MO (5 ng) into the yolk at the one-to two-cell stage and analyzed for EGFP expression at the shield stage and 24 hpf.

### Whole-Mount Immunofluorescence

After fixation in 4% paraformaldehyde and storage in methanol at −20°C, embryos were washed 4 times in PBS containing 1% DMSO, 0.5% Triton X-100, followed by two washes in PBS containing 1% DMSO, 1% Triton X-100, 0.2% BSA and 5% normal goat serum (buffer B). Whole embryos were incubated overnight at 4°C in a 1∶500 dilution of affinity-purified anti-Pacsin3 in buffer B and then washed extensively with several changes of buffer B over 2–3 h. A 1∶500 dilution of Alexa488-congugated anti-rabbit IgG, preadsorbed on fixed zebrafish embryos, was added and incubated overnight at 4°C. After thorough washing with buffer B, the embryos were incubated in Hoechst 33258 stain before mounting for confocal microscopy. Vital staining of live 24 hpf embryos with BODIPY-TR methyl ester was as described [88].

### Transmission EM Analysis

Dechorionated 24 hpf embryos, previously injected with 5 ng of either control or *pacsin3* MO, were fixed in 2.5% glutaraldehyde in PBS and prepared for thin-section EM analysis by standard procedures. Briefly, after post-fixation with 1% osmium tetroxide and 1% potassium ferricyanide for 1 h, the samples were dehydrated in graded alcohols followed by propylene oxide. After embedding in Epon and curing at 60°C, 70-nm thick cross sections of the trunk above the yolk ball were cut and analyzed on a Jeol 1011CX transmission EM.

### Confocal Microscopy

Dechorionated embryos were embedded in 1% low melting point agarose in E3 medium on glass-bottomed MatTech dishes with the dorsal side oriented toward the coverslip. Images were acquired on an Olympus Fluovew1000 instrument using an UplanSapo 20X (NA 0.75) or an UPlanFLN 40x (NA 1.3) oil objective. Data was acquired using the FV10-ASW software and, were necessary, whole TIFF files were minimally adjusted for contrast or brightness and then cropped in Photoshop and assembled in Freehand or Illustrator.

### Transfection and Immunoblotting

HeLa SS6 cells [89] were cultured in DME supplemented with 10% FCS and 2 mM L-glutamine at 37°C in a humidified atmosphere with 5% CO_2_. pCS2+ encoding wild-type Syndapin or various mutants (500 ng) was transfected into cells plated in six-well dishes using Lipofectamine 2000 as detailed elsewhere [90]. After 24 h, cells were collected by trypsinization, and washed cell pellets lysed directly in boiling SDS sample buffer. Equal aliquots of the whole cell lysates were resolved by SDS-PAGE and transferred to nitrocellulose. After blocking in 5% skim milk in TBS, 0.1% Tween 20, blots were probed with either anti-Syndpin antiserum or mAb E7, followed by ECL-type detection.

### Live-Cell Imaging

For live imaging of notochord cells within mGFP- or mcRFP-morphant embryos, the chorion was first removed with forceps at 90% epibody/bud stage and embryos transferred to a custom acrylic chamber filled with E3 solution. Embryos were positioned dorsal side down for confocal imaging on an inverted microscope stage. Glass coverslip fragments secured in place with high vacuum grease held the embryo in place. Chambers were mounted on the stage of an inverted compound microscope (Leica Microsystems) and images collected from a computer controlled SP6 laser scan head. Time-lapse sequences of a single confocal optical section through the notochord were collected with an HCX plan APO 20x (NA 0.7) objective at 1 min intervals using the Leica application suite Advanced Fluorescence software. Any processing or adjustments to the intensity levels were applied equally to all images within a set using Metamorph.

## Supporting Information

Figure S1 Phylogenetic conservation within the PACSIN EFC domain. Multiple sequence alignment of PACSIN homologues used to construct the surface representation of Syndapin shown in Fig. 1E. Residues are colored by identity (dark green), high conservation (green), medium conservation (light green) and no conservation (white). Shown are the protein sequences for *Drosophila melanogaster* Syndapin NP_788697 (Dm_Syndapin); *Danio rerio* Pacsin3 Zgc:56324 (Dr_Pacsin3); *Mus musculus* Pacsin 3 NP_083009.1 (Mm_Pacsin3); *Rattus norvegicus* Pacsin 3 NP_001009966.1 (Rn_Pacsin3); *Homo sapiens* PACSIN 3 NP_057307.2 (Hs_Pacsin3); *Gallus gallus* PACSIN 3 NP_001038117.1 (Gg_Pacsin3); *Xenopus laevis* PACSIN 3 NP_001086374.1 (Xl_Pacsin3); *Homo sapiens* PACSIN 2 NP_009160.2 (Hs_Pacsin2); *Mus musculus* Pacsin 2 NP_035992.1 (Mm_Pacsin2); *Bos taurus* PACSIN 2 NP_001039933.1 (Bt_Pacsin2); *Rattus norvegicus* Pacsin 2 NP_570096.2 (Rn_Pacsin2); *Xenopus laevis* PACSIN 2 NP_001081950.1 (Xl_Pacsin2); *Gallus gallus* PACSIN 2 NP_990420.1 (Gg_Pacsin2); *Danio rerio* Pacsin2 NP_996952.1 (Dr_Pacsin2); *Danio rerio* Pacsin1 NP_001028900.1 (Dr_Pacsin1); *Bos taurus* PACSIN 1 NP_001094571.1 (Bt_Pacsin1); *Rattus norvegicus* Pacsin 1 NP_058990.1 (Rn_Pacsin1); *Mus musculus* Pacsin 1 EDL22546.1 (Mm_Pacsin1); *Xenopus laevis* Pacsin 1 NP_001087407.1 (Xl_Pacsin1); *Homo sapiens* PACSIN 1 NP_065855.1 (Hs_Pacsin1) and *Anopheles gambiae* Pacsin XP_001689033.1 (Ag_Pacsin).
(9.22 MB TIF)

Figure S2 Variability in dimer packing angles in EFC- and BAR-domain proteins. Ribbon diagrams of FCHO2, CIP4, amphiphysin, endophilin, and IRSp53 dimers (one monomer colored green and the other magenta). The apparent radius of curvature (in nm) of the EFC/F-BAR structures of FCHO2 and CIP4, the N-BAR structures of amphiphysin and endophilin, and the I-BAR of IRSp53 is indicated.
(1.83 MB TIF)

Figure S3 Syndapin EFC domain-liposome interactions. (A) Syndapin EFC domain mutant binding to synthetic liposomes. Coomassie-blue stained gels of aliquots of supernatant (S) and pellet (P) fractions from sedimentation assays are shown. (B) Syndapin EFC domain binding to PtdSer containing liposomes. A three-times excess of PtdSer (30%) is bound less effectively than are PtdIns(4,5)P_2_ (10%) containing liposomes. (C) The syndapin EFC domain binds to a similar extent to PtdIns(3)P, PtdIns(3,5)P_2_, PtdIns(4,5)P_2_ and PtdIns(3,4,5)P_3_, but the epsin 1 ENTH domain does not bind PtdIns(3)P. This indicates that the interaction of the Syndapin EFC domain with liposomes is largely via general electrostatics and not strongly stereospecific. (D–F) Negatively-stained transmission electron micrographs of liposome tubulation assays with the Syndapin EFC domain. Folch lipid liposomes alone (panel D), Folch liposomes plus the Syndapin EFC domain (panel E), Folch liposomes plus Syndapin EFC 5K→E mutant (panel F). Scale bar  = 100 nm. Notice that the wild-type Syndapin EFC domain generates both broad (∼80 nm) and narrow (∼20 nm) diameter tubules as well as low levels of small spherical structures that appear to be vesicles. (G) Sensogram traces from assays using PtdIns(4,5)P_2_-containing 200 nm synthetic liposomes immobilized on an L1 chip. The indicated concentration of the *Drosophila* Syndapin EFC domain (1–304; WT), Syndapin (1–304) LMKK→GAAA mutant, epsin 1 ENTH domain or AP-2 core were flowed over the liposomes followed by washing. The derived equilibrium dissociation constant (*K*_D_) values are: 88 nM for the wild-type Syndapin EFC domain, 1.2 µM for the Syndapin LMKK→GAAA mutant, 590 nM for the epsin 1 ENTH domain and 7.3 µM for the heterotetrameric AP-2 core.
(9.77 MB TIF)

Figure S4 Phenotypic range with 5 ng *pacsin3* MO. (A–B) Gross morphology of embryos within the chorion at 24 hpf after injection of 5 ng control MO and 50 pg GFP cRNA at the one- to two-cell stage. Bar  = 250 µm. (C–D) Gross morphology of embryos within the chorion at 24 hpf after injection of 5 ng *pacsin3* MO and 50 pg GFP cRNA at the one- to two-cell stage. Red arrows indicate obvious morphological abnormalities in the morphant embryos. Note too the generally reduced anterioposterior axial length in the pacsin3 MO-injected embryos.
(7.89 MB TIF)

Figure S5 Overexpression of Syndapin mRNA in a wild-type background. (A–I) Representative images of dechorionated 24 hpf embryos after injection of 50 pg capped mRNA encoding GFP (A), or GFP together with 25 pg Drosophila melanogaster (Dm) Syndapin wild type (WT) (B), Dm Syndapin (1–304) (C), Dm syndapin (1–422) (D), Dm Syndapin 5K→E (E), Dm Syndapin LMKK→GAAA (F), Dm Syndapin LMK→GAA (G), Dm Syndapin LM→GA (H), Dm Syndapin LM→GA + KK→AA (I). Notice that at this concentration none of the cRNA injections cause any obvious morphological defects. Bar  = 250 µm.
(6.23 MB TIF)

Table S1 Data collection and refinement statistics
(0.02 MB DOC)

Table S2 Selectivity of *pacsin3* MO target sequence
(0.02 MB DOC)

Video S1 Mediolateral intercalation in control zebrafish notochord. Embryos at the one-cell stage were injected with 100 pg mRNA encoding mcRFP for membrane expression and 7.5 ng control MO. At 9.0 hpf embryos were dechorionated and mounted in E3 medium for time-lapse confocal imaging of the differentiating notochord. Images were collected at 1 min intervals from 90% epiboly (∼9.5 hpf) for 60 min to the one-somite stage (∼10.5 hpf) using a Leica confocal microscope. The image stack was converted into QuickTime video at 10 frames/sec.
(7.70 MB MOV)

Video S2 Defective mediolateral intercalation in mild *pacsin3* morphant notochord. Embryos at the one-cell stage were injected with 100 pg mRNA encoding mcRFP for membrane expression and 7.5 ng *pacsin3* MO. At 9.0 hpf they were dechorionated and mounted in E3 medium for time-lapse confocal imaging of the differentiating notochord. Images were collected at 1 min. intervals from 90% epiboly (∼9.5 hpf) for 60 min to the one-somite stage (∼10.5 hpf) using a Leica confocal microscope. The image stack was converted into QuickTime video at 10 frames/sec.
(7.44 MB MOV)

Video S3 Defective mediolateral intercalation in zebrafish. Continued imaging of the mcRFP-labeled *pacsin3* morphant embryo in Video S2 for an additional 30 min from the one-somite stage (∼10.5 hpf). Images were acquired at 1 min intervals and the image stack was converted into QuickTime video at 10 frames/sec.
(3.41 MB MOV)

Video S4 Defective mediolateral intercalation in severe *pacsin3* morphant notochord. Embryos at the one-cell stage were coinjected with 100 pg mRNA encoding mcRFP for membrane labeling and 7.5 ng *pacsin3* MO. At 9.0 hpf embryos were dechorionated and mounted in E3 medium for time-lapse confocal imaging of the differentiating notochord. Images were collected at 1 min intervals from 90% epiboly (∼9.5 hpf) for 90 min (∼11 hpf) using a Leica confocal microscope. The image stack was converted into QuickTime video at 10 frames/sec.
(8.59 MB MOV)
