# Supplementary figures and images for: Structural Requirements for PACSIN/Syndapin Operation during Zebrafish Embryonic Notochord Development
Source: PLoS One. 2009 Dec 3;4(12):e8150. doi: 10.1371/journal.pone.0008150 (PMC2780292; doi:10.1371/journal.pone.0008150)

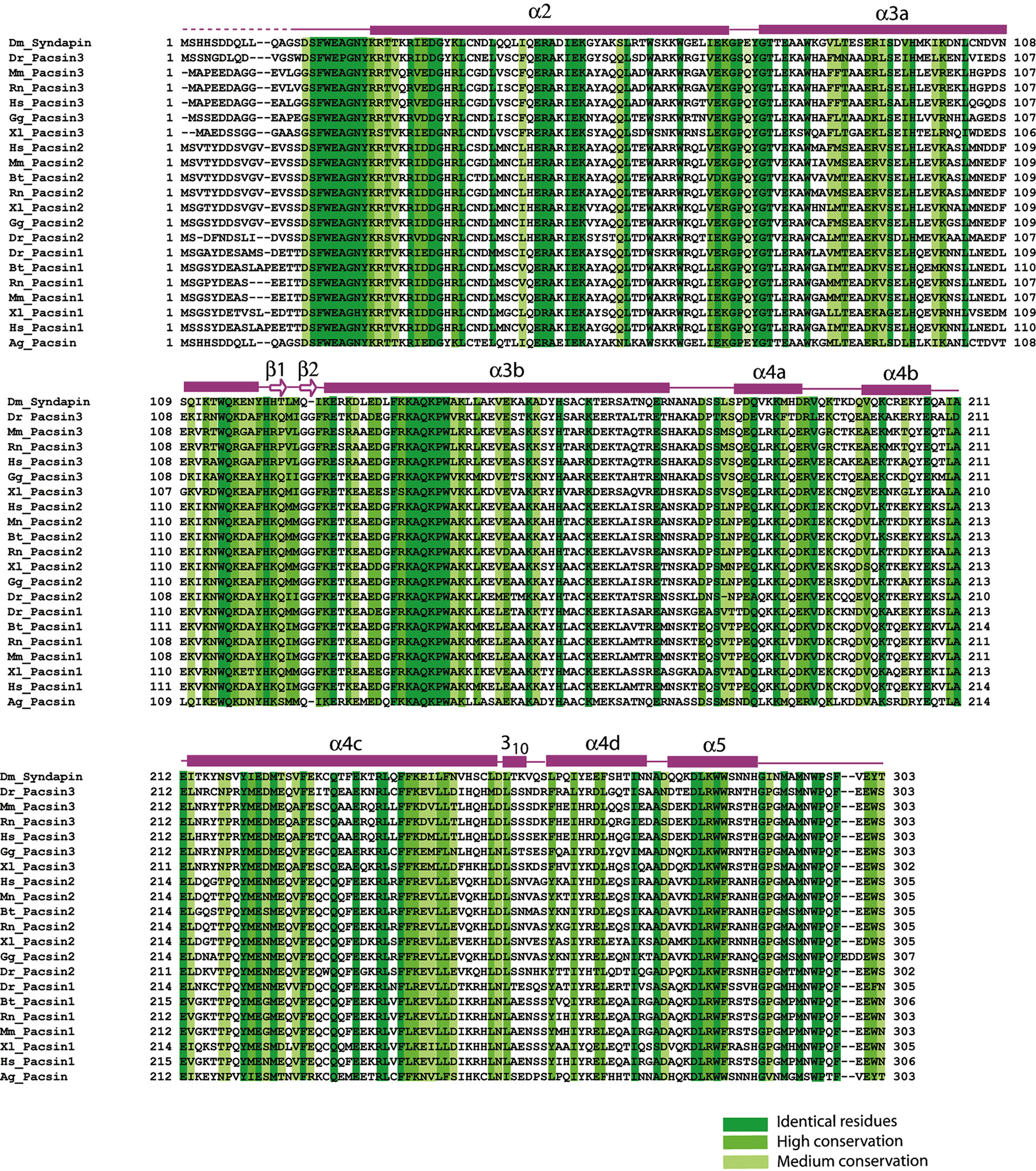

Supplement: Figure S1 — Phylogenetic conservation within the PACSIN EFC domain. Multiple sequence alignment of PACSIN homologues used to construct the surface representation of Syndapin shown in Fig. 1E. Residues are colored by identity (dark green), high conservation (green), medium conservation (light green) and no conservation (white). Shown are the protein sequences for Drosophila melanogaster Syndapin NP_788697 (Dm_Syndapin); Danio rerio Pacsin3 Zgc:56324 (Dr_Pacsin3); Mus musculus Pacsin 3 NP_083009.1 (Mm_Pacsin3); Rattus norvegicus Pacsin 3 NP_001009966.1 (Rn_Pacsin3); Homo sapiens PACSIN 3 NP_057307.2 (Hs_Pacsin3); Gallus gallus PACSIN 3 NP_001038117.1 (Gg_Pacsin3); Xenopus laevis PACSIN 3 NP_001086374.1 (Xl_Pacsin3); Homo sapiens PACSIN 2 NP_009160.2 (Hs_Pacsin2); Mus musculus Pacsin 2 NP_035992.1 (Mm_Pacsin2); Bos taurus PACSIN 2 NP_001039933.1 (Bt_Pacsin2); Rattus norvegicus Pacsin 2 NP_570096.2 (Rn_Pacsin2); Xenopus laevis PACSIN 2 NP_001081950.1 (Xl_Pacsin2); Gallus gallus PACSIN 2 NP_990420.1 (Gg_Pacsin2); Danio rerio Pacsin2 NP_996952.1 (Dr_Pacsin2); Danio rerio Pacsin1 NP_001028900.1 (Dr_Pacsin1); Bos taurus PACSIN 1 NP_001094571.1 (Bt_Pacsin1); Rattus norvegicus Pacsin 1 NP_058990.1 (Rn_Pacsin1); Mus musculus Pacsin 1 EDL22546.1 (Mm_Pacsin1); Xenopus laevis Pacsin 1 NP_001087407.1 (Xl_Pacsin1); Homo sapiens PACSIN 1 NP_065855.1 (Hs_Pacsin1) and Anopheles gambiae Pacsin XP_001689033.1 (Ag_Pacsin). (9.22 MB TIF) [file pone.0008150.s001.tif]

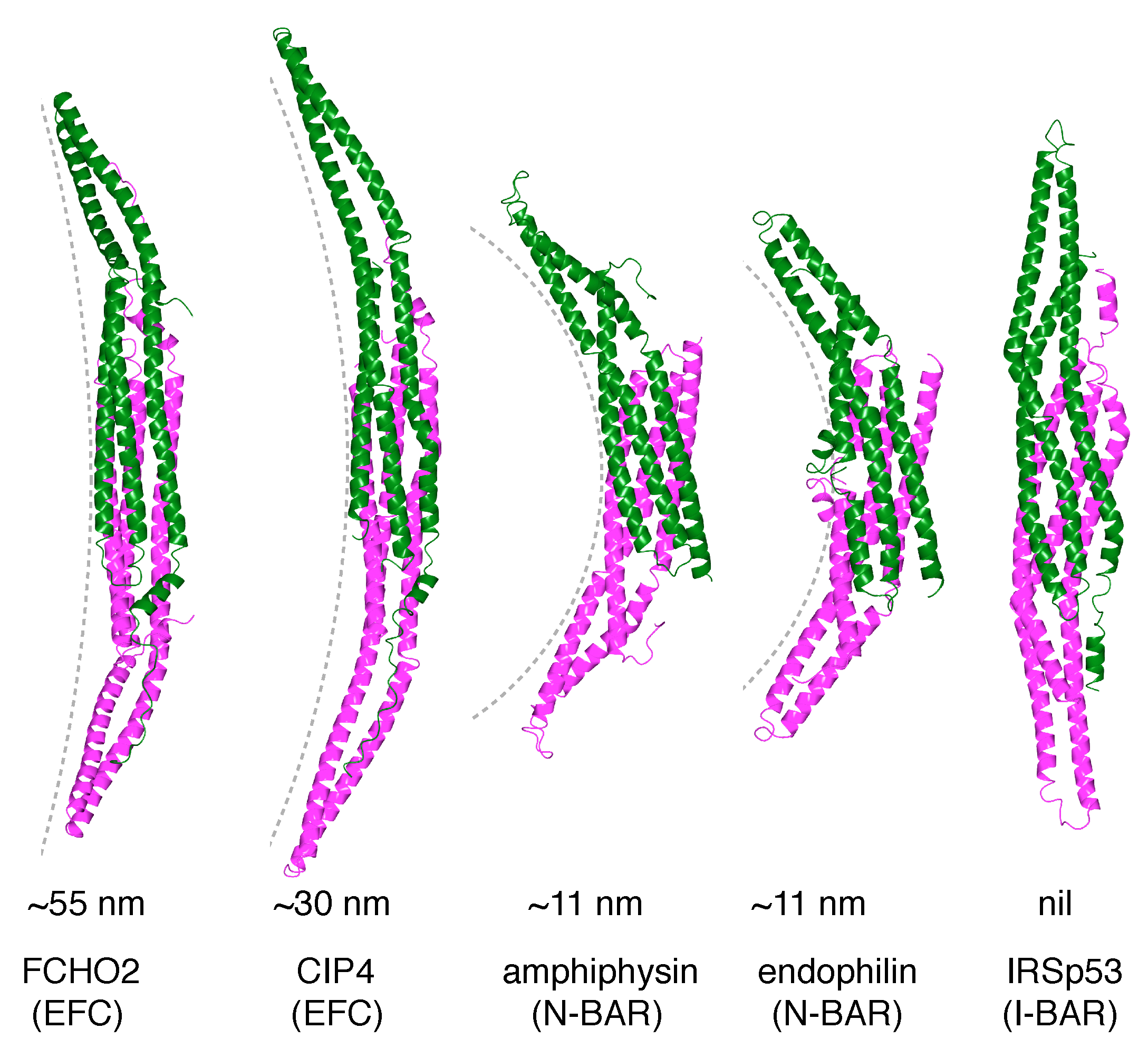

Supplement: Figure S2 — Variability in dimer packing angles in EFC- and BAR-domain proteins. Ribbon diagrams of FCHO2, CIP4, amphiphysin, endophilin, and IRSp53 dimers (one monomer colored green and the other magenta). The apparent radius of curvature (in nm) of the EFC/F-BAR structures of FCHO2 and CIP4, the N-BAR structures of amphiphysin and endophilin, and the I-BAR of IRSp53 is indicated. (1.83 MB TIF) [file pone.0008150.s002.tif]

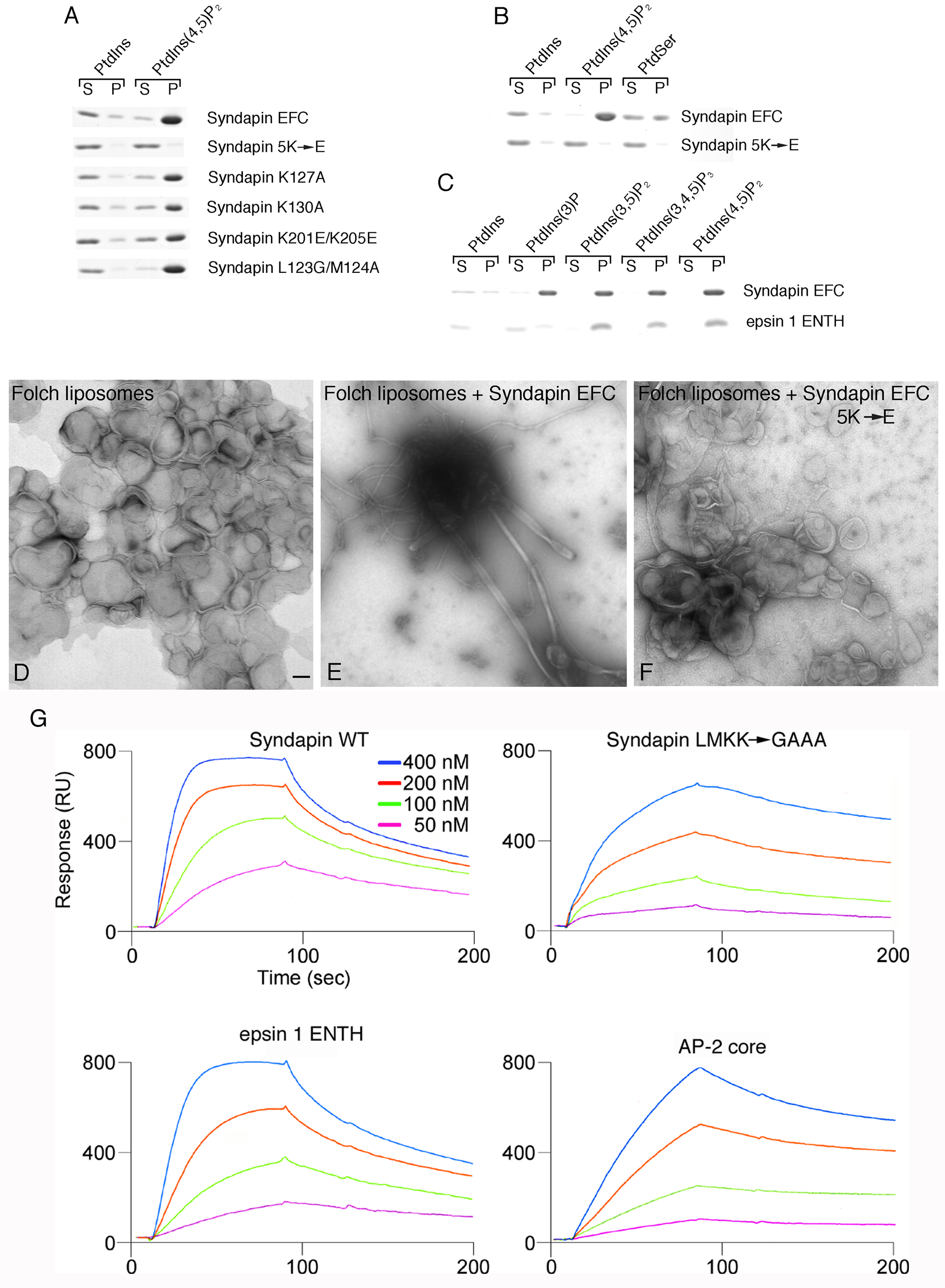

Supplement: Figure S3 — Syndapin EFC domain-liposome interactions. (A) Syndapin EFC domain mutant binding to synthetic liposomes. Coomassie-blue stained gels of aliquots of supernatant (S) and pellet (P) fractions from sedimentation assays are shown. (B) Syndapin EFC domain binding to PtdSer containing liposomes. A three-times excess of PtdSer (30%) is bound less effectively than are PtdIns(4,5)P2 (10%) containing liposomes. (C) The syndapin EFC domain binds to a similar extent to PtdIns(3)P, PtdIns(3,5)P2, PtdIns(4,5)P2 and PtdIns(3,4,5)P3, but the epsin 1 ENTH domain does not bind PtdIns(3)P. This indicates that the interaction of the Syndapin EFC domain with liposomes is largely via general electrostatics and not strongly stereospecific. (D–F) Negatively-stained transmission electron micrographs of liposome tubulation assays with the Syndapin EFC domain. Folch lipid liposomes alone (panel D), Folch liposomes plus the Syndapin EFC domain (panel E), Folch liposomes plus Syndapin EFC 5K→E mutant (panel F). Scale bar = 100 nm. Notice that the wild-type Syndapin EFC domain generates both broad (∼80 nm) and narrow (∼20 nm) diameter tubules as well as low levels of small spherical structures that appear to be vesicles. (G) Sensogram traces from assays using PtdIns(4,5)P2-containing 200 nm synthetic liposomes immobilized on an L1 chip. The indicated concentration of the Drosophila Syndapin EFC domain (1–304; WT), Syndapin (1–304) LMKK→GAAA mutant, epsin 1 ENTH domain or AP-2 core were flowed over the liposomes followed by washing. The derived equilibrium dissociation constant (K D) values are: 88 nM for the wild-type Syndapin EFC domain, 1.2 µM for the Syndapin LMKK→GAAA mutant, 590 nM for the epsin 1 ENTH domain and 7.3 µM for the heterotetrameric AP-2 core. (9.77 MB TIF) [file pone.0008150.s003.tif]

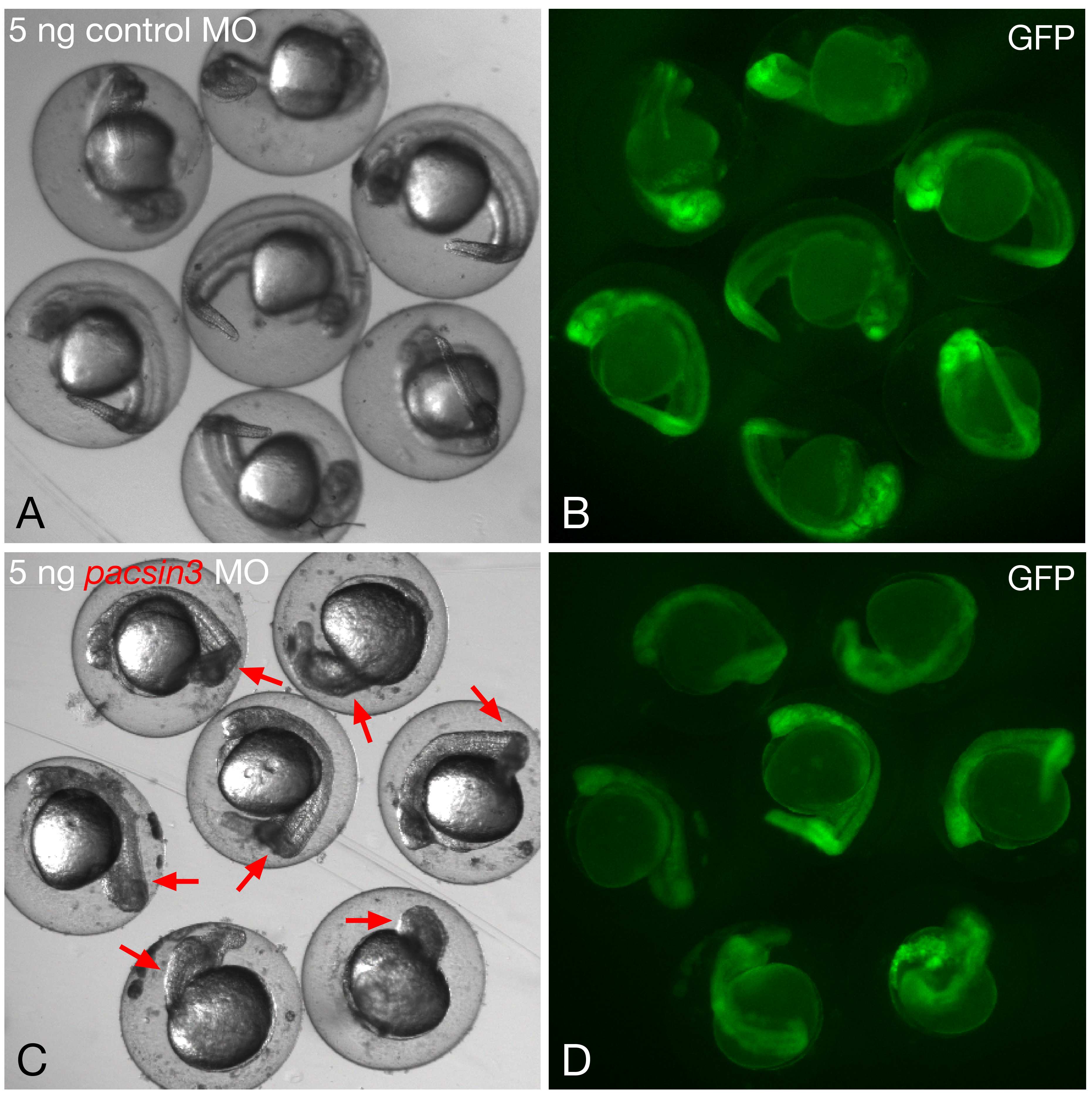

Supplement: Figure S4 — Phenotypic range with 5 ng pacsin3 MO. (A–B) Gross morphology of embryos within the chorion at 24 hpf after injection of 5 ng control MO and 50 pg GFP cRNA at the one- to two-cell stage. Bar = 250 µm. (C–D) Gross morphology of embryos within the chorion at 24 hpf after injection of 5 ng pacsin3 MO and 50 pg GFP cRNA at the one- to two-cell stage. Red arrows indicate obvious morphological abnormalities in the morphant embryos. Note too the generally reduced anterioposterior axial length in the pacsin3 MO-injected embryos. (7.89 MB TIF) [file pone.0008150.s004.tif]

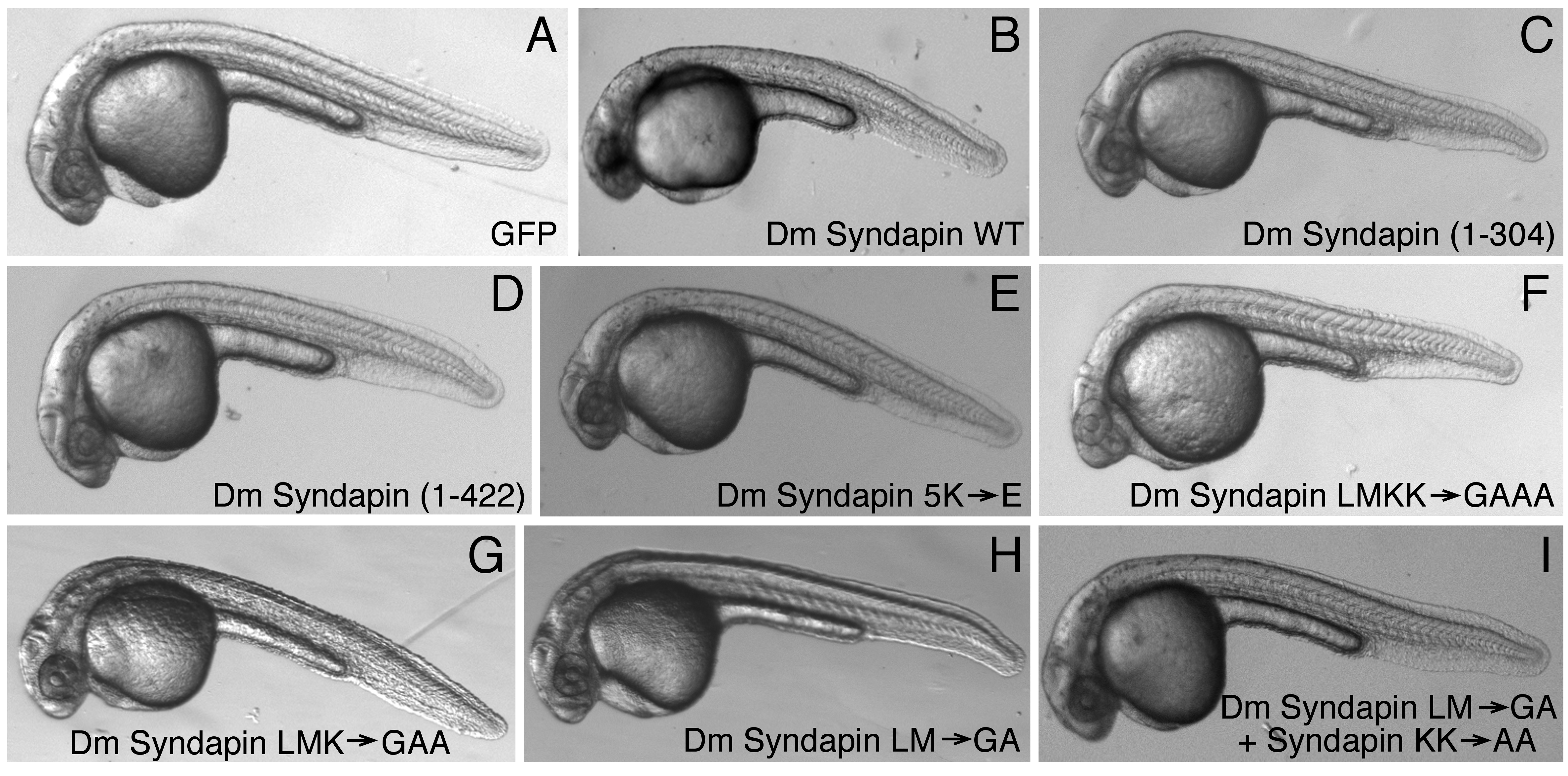

Supplement: Figure S5 — Overexpression of Syndapin mRNA in a wild-type background. (A–I) Representative images of dechorionated 24 hpf embryos after injection of 50 pg capped mRNA encoding GFP (A), or GFP together with 25 pg Drosophila melanogaster (Dm) Syndapin wild type (WT) (B), Dm Syndapin (1–304) (C), Dm syndapin (1–422) (D), Dm Syndapin 5K→E (E), Dm Syndapin LMKK→GAAA (F), Dm Syndapin LMK→GAA (G), Dm Syndapin LM→GA (H), Dm Syndapin LM→GA + KK→AA (I). Notice that at this concentration none of the cRNA injections cause any obvious morphological defects. Bar = 250 µm. (6.23 MB TIF) [file pone.0008150.s005.tif]
