# Supplementary material for: Structural Requirements for PACSIN/Syndapin Operation during Zebrafish Embryonic Notochord Development
Source: PLoS One. 2009 Dec 3;4(12):e8150. doi: 10.1371/journal.pone.0008150 (PMC2780292; doi:10.1371/journal.pone.0008150)
Supplement: Table S1 — Data collection and refinement statistics (0.02 MB DOC) [file pone.0008150.s006.doc]

Table S1. **Data collection and refinement statistics***^a^*

Wavelength (Å) 0.9792

Resolution range (Å) 39.1 - 2.67

*R*merge*^b^* 0.11 (0.38)

*R*meas (within I+/I-)*^c^* 0.12 (0.41)

Mean((I)/sd(I)) 22.2(5.6)

Completeness (%) 99.5

Multiplicity 13.6

Wilson plot B (Å^2^) 56.2

Spacegroup P2_1_2_1_2_1_

Cell dimensions a=62.6 Å, b= 85.5 Å, c=192.9 Å

α=β=γ=90°

**Phasing statistics**

No. sites/no expected 6/8

Figure of merit after SHARP 0.35

Figure of merit after SOLOMON 0.91

**Refinement statistics**

High resolution limit (Å) 2.67

No. of reflections/No. in *R_free_* 29 984/1522

R*_cryst_*/R*_free_^d^* (%) 23.4/25.8

No. atoms protein/water 4 612/103

<B> protein (Å^2^) 43

Rmsd bond lengths (Å) 0.03

Rmsd bond angles (°) 2.4

Ramachandran violations

Most favoured 94.4%

Allowed 5.6%

Disallowed nil

PDB ID 3I2W

*^a^*Numbers in parentheses refer to the highest resolution shell (2.81- 2.67 Å).

*^b^*Rmerge = Σ_hl_ | I_hl_ - <I_h_> | / Σ_hl_ <I_h_>

*^c^*Rmeas = Σ_hl_ [n_h_/( n_h_-1)]^1/2^ I_hl_ - <I_h_> | / Σ_hl_ <I_h_> , where n_h_ is the number of observations of reflection **h**.

*^d^R* = ∑(F_P_-F_calc_)/ ∑F_P_
