# Supplementary material for: Structural Requirements for PACSIN/Syndapin Operation during Zebrafish Embryonic Notochord Development
Source: PLoS One. 2009 Dec 3;4(12):e8150. doi: 10.1371/journal.pone.0008150 (PMC2780292; doi:10.1371/journal.pone.0008150)
Supplement: Table S2 — Selectivity of pacsin3 MO target sequence (0.02 MB DOC) [file pone.0008150.s007.doc]

Table S2. **Selectivity of *pacsin3* MO target sequence**

| gene^a^ | accession | sequence | mismatch |
| --- | --- | --- | --- |
| *pacsin3* MO | - | CAAA**ATG**TCTTCCAACGGTGATCTGC | - |
| *pacsin3* (zgc:56324) | BC046073 | CAAA**ATG**TCTTCCAACGGTGATCTGC | 0 bp |
| *pacsin*1 | BC124238 | CATC**ATG**TCAACGCTTCCGGCAGAGT | 16 bp |
| *pacsin*2 | NM207069 | AGAC**ATG**TCGGATTTCAACGACTCTC | 16 bp |
| zgc:109968 | BC095091 | CACC**ATG**TCTGGCTCCTACGATGAAT | 13 bp |
| zgc:91999 | NM001003498 | GATC**ATG**GCGGATCCTCCAACAGGCC | 20 bp |
| A3KNY2-1 | BC134071 | CATC**ATG**TCAACGCTTCCGGCAGAGT | 16 bp |

^a^The six PACSIN family ortholog genes encoded in the *Danio rerio* genome are listed.
